# Causes of Death Among Infants and Children in the Child Health and Mortality Prevention Surveillance (CHAMPS) Network

**DOI:** 10.1001/jamanetworkopen.2023.22494

**Published:** 2023-07-26

**Authors:** Quique Bassat, Dianna M. Blau, Ikechukwu Udo Ogbuanu, Solomon Samura, Erick Kaluma, Ima-Abasi Bassey, Samba Sow, Adama Mamby Keita, Milagritos D. Tapia, Ashka Mehta, Karen L. Kotloff, Afruna Rahman, Kazi Munisul Islam, Muntasir Alam, Shams El Arifeen, Emily S. Gurley, Vicky Baillie, Portia Mutevedzi, Sana Mahtab, Bukiwe Nana Thwala, Beth A. Tippett Barr, Dickens Onyango, Victor Akelo, Emily Rogena, Peter Onyango, Richard Omore, Inacio Mandomando, Sara Ajanovic, Rosauro Varo, Antonio Sitoe, Miquel Duran-Frigola, Nega Assefa, J. Anthony G. Scott, Lola Madrid, Tseyon Tesfaye, Yadeta Dessie, Zachary J. Madewell, Robert F. Breiman, Cynthia G. Whitney, Shabir A. Madhi

**Affiliations:** 1 ISGlobal–Hospital Clínic, Universitat de Barcelona, Barcelona, Spain; 2 Centro de Investigação em Saúde de Manhiça–CISM, Maputo, Mozambique; 3 ICREA, Pg. Lluís Companys 23, Barcelona, Spain; 4 Pediatrics Department, Hospital Sant Joan de Déu, Universitat de Barcelona, Esplugues, Barcelona, Spain; 5 Consorcio de Investigación Biomédica en Red de Epidemiología y Salud Pública–CIBERESP, Madrid, Spain; 6 Center for Global Health, Centers for Disease Control and Prevention, Atlanta, Georgia; 7 Crown Agents, Freetown, Sierra Leone; 8 World Hope International, Makeni, Sierra Leone; 9 Centre pour le Développement des Vaccins, Ministère de la Santé, Bamako, Mali; 10 Department of Pediatrics, Center for Vaccine Development and Global Health, University of Maryland School of Medicine, Baltimore; 11 International Center for Diarrhoeal Diseases Research, Dhaka, Bangladesh; 12 Department of Epidemiology, Johns Hopkins Bloomberg School of Public Health, Baltimore, Maryland; 13 South African Medical Research Council Vaccines and Infectious Diseases Analytics Research Unit; Faculty of Health Sciences, University of the Witwatersrand, Johannesburg, South Africa; 14 Centers for Disease Control and Prevention–Kenya, Kisumu, Kenya; 15 Kisumu County Department of Health, Kisumu, Kenya; 16 Jomo Kenyatta University of Agriculture and Technology, Juja, Kenya; 17 Kenya Medical Research Institute, Center for Global Health Research, Kisumu, Kenya; 18 Instituto Nacional de Saúde, Ministério de Saúde, Maputo, Moçambique; 19 Ersilia Open Source Initiative; Cambridge, United Kingdom; 20 College of Health and Medical Sciences, Haramaya University, Harar, Ethiopia; 21 Department of Infectious Disease Epidemiology, London School of Hygiene & Tropical Medicine, London, United Kingdom; 22 KEMRI-Wellcome Trust Research Programme, Kilifi, Kenya; 23 Emory Global Health Institute, Emory University, Atlanta, Georgia; 24 Department of Global Health, Rollins School of Public Health, Emory University, Atlanta, Georgia; 25 Wits Infectious Diseases and Oncology Research Institute, Faculty of Health Sciences, University of the Witwatersrand, Johannesburg, South Africa

## Abstract

**Importance:**

The number of deaths of children younger than 5 years has been steadily decreasing worldwide, from more than 17 million annual deaths in the 1970s to an estimated 5.3 million in 2019 (with 2.8 million deaths occurring in those aged 1-59 months [53% of all deaths in children aged <5 years]). More detailed characterization of childhood deaths could inform interventions to improve child survival.

**Objective:**

To describe causes of postneonatal child deaths across 7 mortality surveillance sentinel sites in Africa and Asia.

**Design, Setting, and Participants:**

The Child Health and Mortality Prevention Surveillance (CHAMPS) Network conducts childhood mortality surveillance in sub-Saharan Africa and South Asia using innovative postmortem minimally invasive tissue sampling (MITS). In this cross-sectional study, MITS was conducted in deceased children aged 1 to 59 months at 7 sites in sub-Saharan Africa and South Asia from December 3, 2016, to December 3, 2020. Data analysis was conducted between October and November 2021.

**Main Outcomes and Measures:**

The expert panel attributed underlying, intermediate, and immediate conditions in the chain of events leading to death, based on histopathologic analysis, microbiological diagnostics, clinical data, and verbal autopsies.

**Results:**

In this study, MITS was performed in 632 deceased children (mean [SD] age at death, 1.3 [0.3] years; 342 [54.1%] male). The 6 most common underlying causes of death were malnutrition (104 [16.5%]), HIV (75 [11.9%]), malaria (71 [11.2%]), congenital birth defects (64 [10.1%]), lower respiratory tract infections (LRTIs; 53 [8.4%]), and diarrheal diseases (46 [7.2%]). When considering immediate causes only, sepsis (191 [36.7%]) and LRTI (129 [24.8%]) were the 2 dominant causes. An infection was present in the causal chain in 549 of 632 deaths (86.9%); pathogens most frequently contributing to infectious deaths included *Klebsiella pneumoniae* (155 of 549 infectious deaths [28.2%]; 127 [81.9%] considered nosocomial), *Plasmodium falciparum* (122 of 549 [22.2%]), and *Streptococcus pneumoniae* (109 of 549 [19.9%]). Other organisms, such as cytomegalovirus (57 [10.4%]) and *Acinetobacter baumannii* (39 [7.1%]; 35 of 39 [89.7%] considered nosocomial), also played important roles. For the top underlying causes of death, the median number of conditions in the chain of events leading to death was 3 for malnutrition, 3 for HIV, 1 for malaria, 3 for congenital birth defects, and 1 for LRTI. Expert panels considered 494 of 632 deaths (78.2%) preventable and 26 of 632 deaths (4.1%) preventable under certain conditions.

**Conclusions and Relevance:**

In this cross-sectional study investigating causes of child mortality in the CHAMPS Network, results indicate that, in these high-mortality settings, infectious diseases continue to cause most deaths in infants and children, often in conjunction with malnutrition. These results also highlight opportunities for action to prevent deaths and reveal common interaction of various causes in the path toward death.

## Key Points

**Question:**

Can the investigation of causes of childhood death in high-mortality settings be improved by the use of postmortem minimally invasive tissue sampling?

**Findings:**

This cross-sectional study presents data on 632 postneonatal deaths (age 1-59 months) from 7 sub-Saharan and Asian high-mortality sites adequately characterized through histopathologic analysis, microbiological diagnostics, clinical data, and verbal autopsies. Malnutrition, HIV, malaria, congenital birth defects, lower respiratory tract infections, and diarrheal diseases were the most common underlying causes of childhood deaths, an infection was present in the causal chain in 86.9% of the cases, and 82.3% of deaths were deemed potentially preventable by the expert panels evaluating them.

**Meaning:**

The results of this study demonstrate the potential of the minimally invasive tissue sampling tool to reliably investigate causes of child deaths and highlight the significant role of infections in high-child-mortality settings, providing concrete opportunities for action to enhance child survival.

## Teaser

This cross-sectional study investigates the principal causes of child mortality by exploring 4 years of cause-of-death cumulative data from 7 mortality surveillance sentinel sites in Africa and Asia.

## Introduction

The number of deaths of children younger than 5 years has been steadily decreasing worldwide, from more than 17 million annual deaths in the 1970s to an estimated 5.3 million in 2019 (with 2.8 million deaths occurring in those aged 1-59 months [ie, 53% of all deaths in children aged <5 years]).^1^ However, such impressive progress is nuanced because significant differences remain among geographic regions.^2^ Reductions in child mortality have been comparatively modest in low- and middle-income countries (LMICs), which now account for up to 99% of all global child deaths,^3,4^ a striking reminder of the many inequities that impair global health.

Our current understanding of what is causing deaths among children today in LMICs—and thus globally—is blurred by the inadequacy and poor reliability of the source data used to construct mortality burden estimates. In LMICs, the 2 methods most widely used are the verbal autopsy and premortem clinical records, both of which have repeatedly shown insufficient robustness and granularity to identify causes of death (CODs) at the individual level without major errors.^5,6,7^ An additional problem relates to the way CODs have traditionally been summarized and described for statistical and comparison purposes: with the focus on underlying CODs (ie, “the disease or injury that initiated the train of morbid events leading directly to death”^8^). Although the underlying COD is important, it is often just the first step of a chain of events (causal chain) that includes 1 or more morbid (also termed *intermediate*) conditions and a final event, typically known as the immediate COD. Analyzing major causes of child mortality only using the underlying COD assumes that a child can only die of 1 cause and that a hierarchical relationship among conditions leading to death exists, when in fact there may be interactions. Such an oversimplification fails to recognize the multidimensional and complex nature of the process.^9^

The Child Health and Mortality Prevention Surveillance (CHAMPS) Network was established in 2016 as a long-term collaborative platform to conduct standardized, comprehensive, and high-quality surveillance for causes of child mortality (including stillbirths) in high-mortality, sentinel sites in sub-Saharan Africa and Asia.^10^ Core surveillance activities include the use of the innovative postmortem minimally invasive tissue sampling (MITS) approach for COD investigation,^11^ which is more reliable than verbal autopsy and clinical records investigation^7,12^ and more socially and culturally acceptable than complete diagnostic autopsies.^13^ The accumulation of cases from CHAMPS sentinel sites is providing a more robust backbone to support future child mortality statistics. In this cross-sectional study, we aimed to investigate the principal causes of child mortality by exploring 4 years of COD cumulative data from 7 CHAMPS sites in the postneonatal period (1-59 months of age), with a special emphasis on illustrating the complexities of causal chains and the role of infectious diseases and providing data-driven recommendations on approaches to enhance child survival.

## Methods

### Mortality and Demographic Surveillance

Criteria required to become a CHAMPS mortality surveillance site, the mortality surveillance strategy and standardized approaches, and the detailed inclusion criteria for enrollment and MITS have been previously described.^14^ In brief, stillbirths and children younger than 5 years at the time of death and residing within the catchment area of a CHAMPS site were eligible for enrollment and MITS if identified within 24 hours of occurrence (36 hours if the body was preserved through refrigeration). In all cases, a written informed consent signed by legal guardians was required to allow the standardized collection of clinical data from the child and mother, tissues from key organs and body fluid samples (through MITS), pictures (for morphologic identification), and anthropometric measurements and to conduct a verbal autopsy.^15^ This cross-sectional study focuses on postneonatal deaths (≥28 days to <60 months), given the marked differences in underlying causes in the neonatal period compared with the postneonatal period, in children aged 1 to 59 months at 7 sites in sub-Saharan Africa and South Asia from December 3, 2016, to December 3, 2020. We defined community deaths as any death occurring prior to arrival at a health facility, whereas facility deaths included those occurring in a child who arrived at the health facility alive. Ethics committees or institutional review boards overseeing protocol implementation at each site and at Emory University approved the generic and site-specific protocols as appropriate. The Centers for Disease Control and Prevention (CDC) relied on Emory University to review the overall protocol and perform appropriate site ethical review when the CDC staff was directly engaged at the site. Protocols are available on the CHAMPS website.^16^ This study followed the Strengthening the Reporting of Observational Studies in Epidemiology (STROBE) reporting guideline.

### Specimen and Data Collection

The MITS protocol^11^ included the non–imaging-guided postmortem collection of blood, cerebrospinal fluid, stool specimens, and oropharyngeal/nasopharyngeal swabs together with the sampling of key organs (brain, lungs, and liver) using a fine-needle biopsy-like technique. Microbiological evaluation of samples included the use of on-site culture of blood and cerebrospinal fluid and proactive screening with molecular methods (TaqMan Array cards; Thermo Fisher Scientific) configured by syndrome and customized to include a large number of pathogens of significance in young children (including 57 bacteria, 48 viruses, 8 parasites, and 3 fungi).^17^ Specific additional screening was conducted for malaria, HIV, and tuberculosis. An initial histopathologic assessment was conducted locally at each site and complemented with specific stains, immunohistochemistry, and further molecular methods if deemed necessary at the central pathology laboratory at the Infectious Diseases Pathology Branch of the CDC.^18^

### COD Determination

All available data, including sociodemographic, clinical, laboratory data (microbiological and histopathologic), pictures of the body, and the verbal autopsy were compiled into standardized DeCoDe (Determination of Cause of Death) packages, made available to a panel of local experts, including pediatricians, obstetricians, public health specialists, pathologists, and microbiologists. The panel discussed each case, reaching (with or without full consensus^19^) a COD diagnosis, which necessarily included an underlying COD and, if applicable, intermediate (or morbid) conditions and an immediate COD, thus establishing the most likely chain of events leading to death. Time ordering of events was established, whenever possible, by using the narrative information provided by the clinical history and/or the verbal autopsy. *International Statistical Classification of Diseases and Related Health Problems, Tenth Revision* (*ICD-10*) nomenclature was used to code diagnoses, following the prespecified CHAMPS specific diagnosis standards,^19^ and maternal diagnoses included where applicable.

### Statistical Analysis

Data analysis was performed from October to November 2021. Data analyses in this manuscript are restricted to cases for which a DeCoDe panel had completed a COD determination, irrespective of whether a known COD was identified. Given that each death could have up to several intermediate causes, we present details on the median number of causes and have opted for pooling intermediate causes in certain analyses. Data were examined using frequency distributions, and χ^2^ analysis was used for comparisons of categorical variables. Statistical testing was 2-sided at the *P* < .05 significance level.

## Results

### Screening and Enrollment

Mortality surveillance identified 2332 deceased children aged 28 days to younger than 5 years, of whom 1004 were considered eligible for CHAMPS recruitment and postmortem investigations, and 721 (71.8%) underwent MITS. Of these, 632 deaths (87.7%) had a COD determined through a DeCoDe panel on time for the analysis; therefore, MITS was performed in 632 deceased children (mean [SD] age at death, 1.3 [0.3] years; 342 [54.1%] male and 290 [45.9%] female) (Table 1). The distribution of recruitment among CHAMPS sites is shown in eFigure 1 in Supplement 1.

**Table 1.** Selected Characteristics of Deaths Among Children Aged 28 Days Through 59 Months With Complete Cause of Death Determination, by Site and Combined for the CHAMPS Network, December 2016 to December 2020^a^

Characteristic	South Africa (n = 173)	Mozambique (n = 101)	Kenya (n = 195)	Ethiopia (n = 20)	Sierra Leone (n = 89)	Mali (n = 51)	Bangladesh (n = 3)	Total (N = 632)
Age group								
Infant (28 d to <12 mo)	112 (64.7)	40 (39.6)	109 (55.9)	9 (45.0)	32 (36.0)	27 (52.9)	2 (66.7)	331 (52.4)
Child (12 to <60 mo)	61 (35.3)	61 (60.4)	86 (44.1)	11 (55.0)	57 (64.0)	24 (47.1)	1 (33.3)	301 (47.6)
Religion of primary caretaker								
Muslim	1 (0.6)	6 (5.9)	2 (1.0)	20 (100)	71 (79.8)	51 (100)	3 (100)	154 (24.4)
Christian	116 (67.0)	34 (33.7)	181 (92.8)	0	17 (19.1)	0	0	348 (55.0)
African traditional religion	1 (0.6)	9 (8.9)	10 (5.2)	0	0	0	0	20 (3.2)
Other or no religion or unknown	55 (31.8)	52 (51.5)	2 (1.0)	0	1 (1.1)	0	0	110 (17.4)
Sex of deceased								
Female	77 (44.5)	37 (36.6)	91 (46.7)	8 (40.0)	45 (50.6)	31 (60.8)	1 (33.3)	290 (45.9)
Male	96 (55.5)	64 (63.4)	104 (53.3)	12 (60.0)	44 (49.4)	20 (39.2)	2 (66.7)	342 (54.1)
Time from death to MITS procedure, h^b^								
≤24	100 (57.8)	87 (86.1)	149 (76.4)	20 (100)	89 (100)	51 (100)	3 (100)	499 (79.1)
>24	73 (42.2)	13 (13.9)	46 (23.6)	0	0	0	0	132 (20.9)
Location and timing of death								
Facility, ≤24 h after admission	37 (21.4)	49 (48.5)	65 (33.3)	4 (20.0)	36 (40.5)	21 (41.2)	0	212 (33.5)
Facility, >24 h after admission	112 (64.7)	37 (36.6)	48 (24.6)	4 (20.0)	35 (39.3)	5 (9.8)	3 (100)	244 (38.6)
Community	24 (13.9)	15 (14.9)	82 (42.1)	12 (60.0)	18 (20.2)	25 (49.0)	0	176 (27.9)
Duration of hospitalization, median (IQR), h^c^	122 (25-930)	20 (5-108)	17 (7-72)	41 (0-122)	22 (7-75)	3 (0-13)	62 (46-183)	30 (7-160)
Exposure to HIV-infected mother	66 (38.2)	45 (44.6)	56 (28.7)	0	5 (5.6)	4 (7.8)	0	176 (27.8)
HIV infected	21 (12.1)	22 (21.8)	27 (13.8)	0	5 (5.6)	4 (7.8)	0	79 (12.5)
Low birth weight^d^	32 (48.4)	7 (24.1)	20 (18.0)	0	3 (15.8)	6 (20.7)	0	68 (26.8)
Severe underweight (WAZ < −3 SDs)^e^	73 (44.5)	46 (46.9)	90 (46.4)	14 (82.4)	34 (38.2)	21 (41.2)	2 (66.7)	280 (45.5)
Severe stunting (HAZ < −3 SDs)^f^	57 (33.1)	42 (42.9)	40 (20.5)	11 (64.7)	14 (15.7)	12 (23.5)	0	176 (28.2)
Severe wasting (WHZ < −3 SDs)^g^	47 (32.6)	33 (34.7)	82 (44.1)	7 (38.9)	43 (49.4)	28 (54.9)	3 (100)	243 (41.6)

Abbreviations: HAZ, height-for-age *z* score; MITS, minimally invasive tissue sampling; WAZ, weight-for-age *z* score; WHZ, weight-for-height *z* score.

^a^ Data are presented as number (percentage) unless otherwise indicated.

^b^ N = 631.

^c^ For those dying at a health care facility.

^d^ N = 254.

^e^ N = 616.

^f^ N = 625.

^g^ N = 584.

Deaths occurred within the community (176 [27.8%]) or within associated health facilities (456 [72.2%]). Of 456 in-hospital deaths, 212 (46.5%) occurred within the first 24 hours of admission, and 361 (79.2%) of all MITS were conducted within 24 hours of death (median [IQR] time, 13.3 [6.3-21.5] hours). Of all 632 decedents, 176 (27.8%) were children born of mothers known to be HIV positive (ie HIV exposed), and 79 deaths (12.5%) were confirmed as HIV-1 infections, with 70 deaths (88.6%) from HIV infections coming from only 3 sites (Kenya [n = 27], Mozambique [n = 22], and South Africa [n = 21]). Of 186 deaths with known birth weight, 68 children (26.8%) had low birth weight. Furthermore, 280 of 616 deceased children (45.5%) were considered severely underweight (weight-for-age *z* score < −3 SDs), 176 of 625 (28.2%) were considered severely stunted (height-for-age *z* score < −3 SDs), and 243 of 584 (41.6%) were considered severely wasted (weight-for-height *z* score < −3 SDs).

### CODs and Causal Pathways

**Figure 1.**
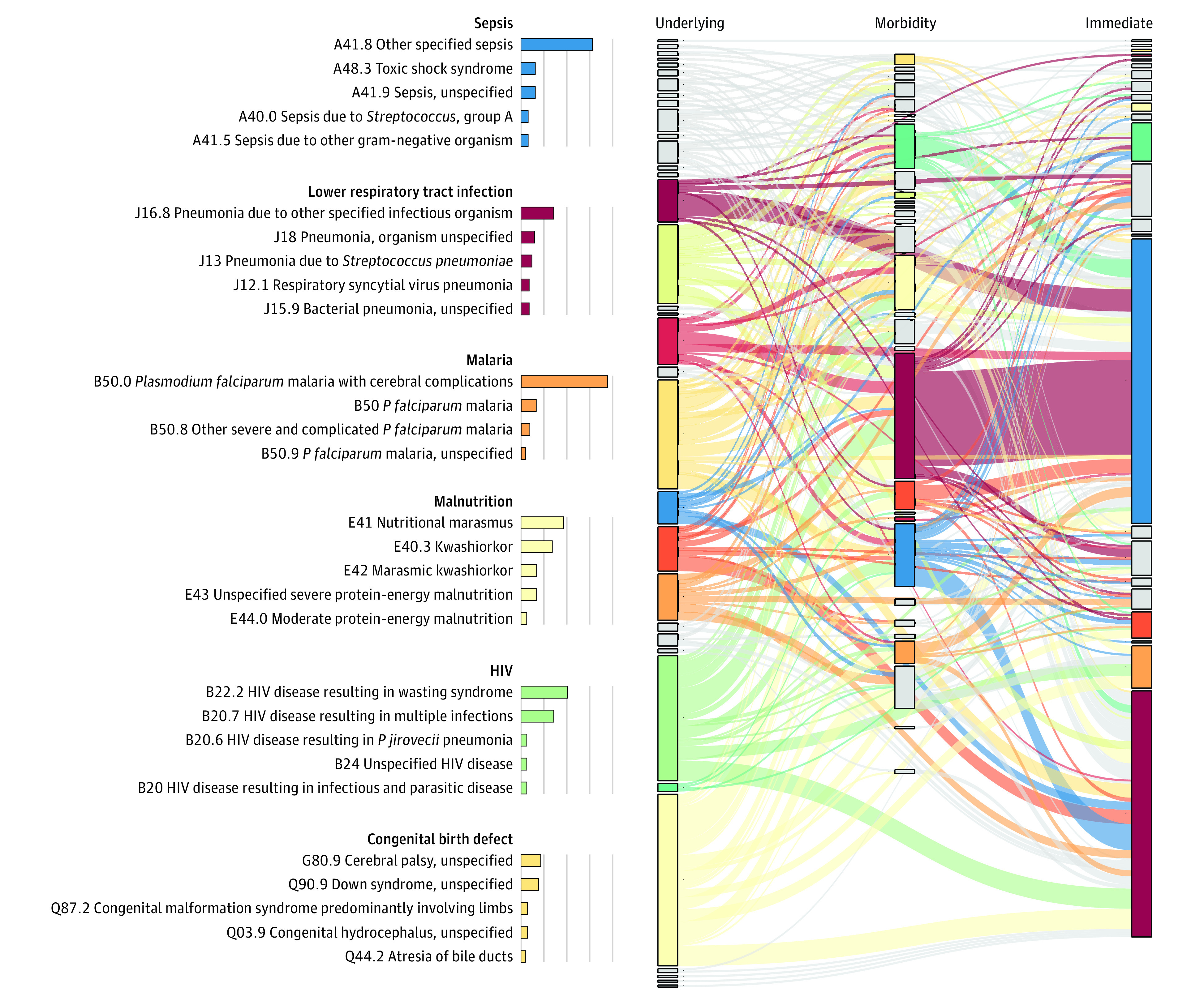
Sankey Diagram Illustrating the Pathways Linking the Different Steps (Underlying Causes, Pooled Morbid or Intermediate Conditions, and Immediate Causes) of the Causal Pathway Leading to Death For the major causes, a more detailed breakdown of their characteristics is shown. Note that only cases with 2 or more events are shown in the Sankey diagram (ie, to avoid confusion); cases with only 1 event (ie, the underlying cause is the immediate cause) are not depicted. Data supporting this figure are available in eTable 5 in Supplement 1. As an example, in the left column (underlying causes), the different diagnoses related to malnutrition (E4* *International Statistical Classification of Diseases and Related Health Problems, Tenth Revision* [*ICD-10*] codes, pale yellow color) represent the largest proportion of underlying causes, linking to a myriad of morbid causes, but in particular with lower respiratory infections and sepsis (as morbid or immediate cause of death).

The DeCoDe panels could not attribute any cause of death for 15 (10 community and 5 facility deaths) of 632 enrolled decedents (2.4%). A total of 156 deaths (24.7%) were determined to be due to a single cause, with 461 (72.9%) having 2 or more conditions identified in the causal chain. The median number of conditions for both infants (aged 28 days to <12 months) and older children was 2 (range, 1-7) for both groups. For the 5 most common underlying causes, the median number of events in the causal chain was 3 (range, 1-6) for malnutrition, 3 (range, 1-5) for HIV, 1 (range, 1-4) for malaria, 3 (range, 1-5) for congenital birth defects, and 1 (range, 1-5) for LRTI (eTable 1 in Supplement 1). The median number of causes identified in the pathway was 2 (range, 1-7) for deaths occurring in the community, 2 (range, 1-5) for deaths occurring 24 hours or less of admission to a facility, and 3 (range, 1-7) for those dying more than 24 hours after admission (eTable 2 in Supplement 1). The number of causes varied by site (eTable 3 in Supplement 1) and according to underlying cause, with malaria (n = 71 deaths) and LRTI (n = 53 deaths) most often having no other causes in the chain of events (eTable 4 in Supplement 1). For the 632 cases, the full sequence of events can be interactively consulted in a sunburst diagram^20^ (eFigure 2 in Supplement 1). Causal chains were often complex, as illustrated in Figure 1 by a Sankey diagram linking the different underlying CODs with intermediate (all pooled together under the term *morbidity*) CODs (eTable 5 in Supplement 1).

**Figure 2.**
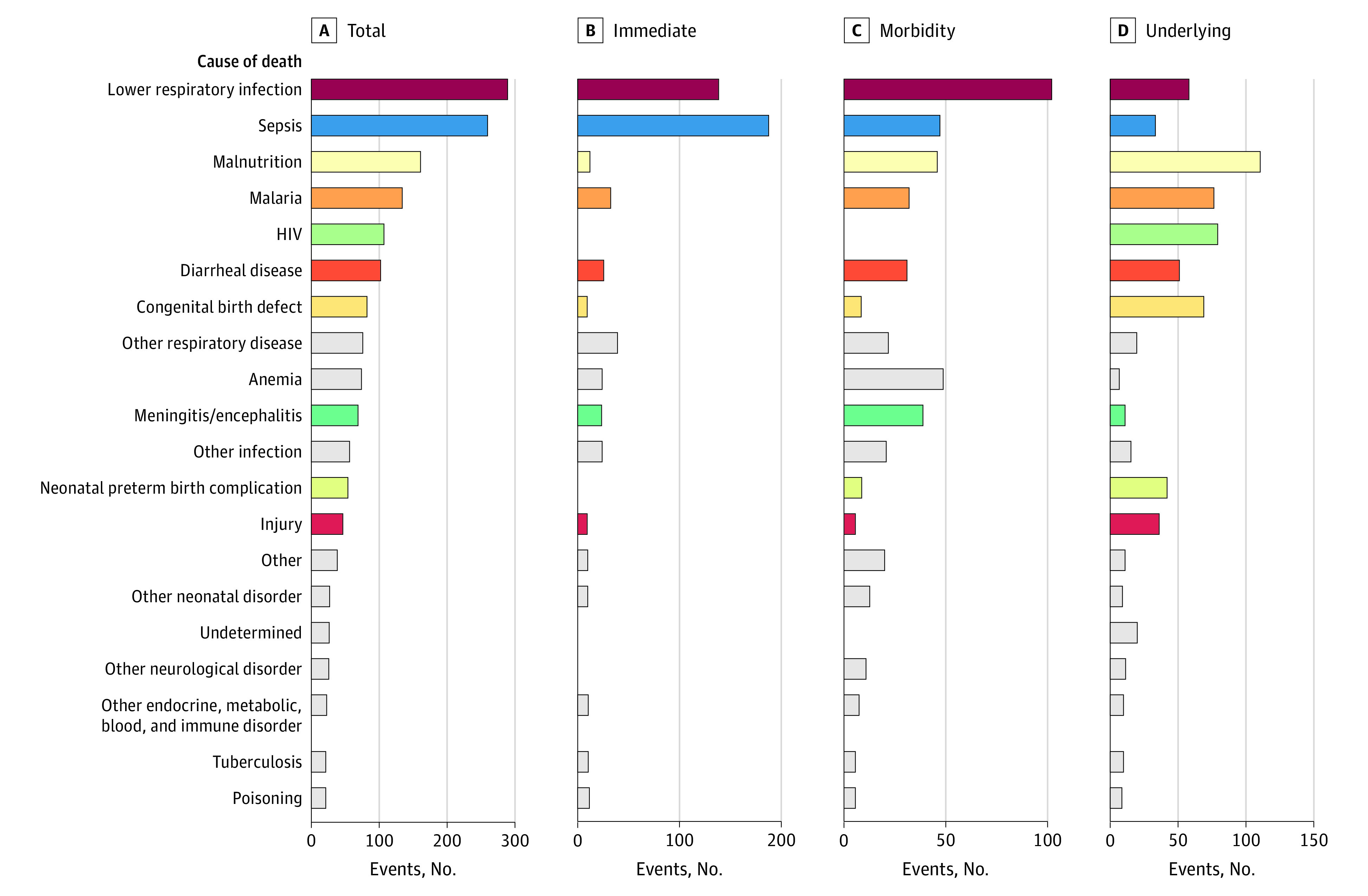
Frequency of Occurrence of the Most Common 20 Diagnoses According to the Position in the Chain of Events Data are shown for total (any location in causal chain), underlying, intermediate or comorbid (pooled), and immediate causes of death. Note that x-axis scales differ between the panels. Also note that all deaths are assigned an underlying cause; some may also have 1 immediate cause and from none to multiple intermediate causes (Figure 1).

The most common underlying CODs precipitating the chain of events leading to death included malnutrition (104 cases [16.5%]), HIV/AIDS (75 [11.9%]), malaria (71 [11.2%]), congenital birth defects (64 [10.1%]), LRTI (53 [8.4%]), and diarrheal disease (46 [7.2%]) (Figure 2). When looking only at immediate CODs (461 deaths had an immediate COD in addition to an underlying COD), sepsis (191 [36.7%]) and LRTI (129 [24.8%]) were by far the 2 most common diagnoses. When all steps of the chain of events are considered together, LRTI (280 [44.3%]), sepsis (253 [40.0%]), malnutrition (150 [23.7%]), malaria (123 [19.5%]), and diarrheal diseases (90 [14.2%]) remained the most common CODs in this series. Importantly, maternal conditions were deemed to play a role in the causal chain in 50 of the 632 deaths (7.9%), with maternal HIV (21 deaths) and pregnancy-associated hypertensive disorders (6 deaths) being the 2 most common maternal diagnoses. Maternal factors played a more important role among young infants (aged 1-3 months: 33 of 156 [21.2%]; aged 3-6 months: 8 of 77 [10.4%]) but also affected the older groups (10 of 399 [2.5%]).

Of note, the occurrence and importance of some diagnoses differed significantly depending on whether the death took place at home or within the health care system (eTable 6 in Supplement 1). Lower respiratory tract infections (219 [48.0%] vs 61 [34.7%]), birth defects (62 [13.6%] vs 8 [4.5%]), meningitis/encephalitis (48 [10.5%] vs 9 [5.1%]), and anemia (59 [12.9%] vs 3 [1.7%]) were more frequently diagnosed among in-hospital deaths, whereas sudden infant death syndrome (5 [2.8%] vs 0) and malnutrition were more common (51 [29.0%] vs 99 [21.7%]) in community deaths.

### Contribution of Infections to Postneonatal Deaths

**Figure 3.**
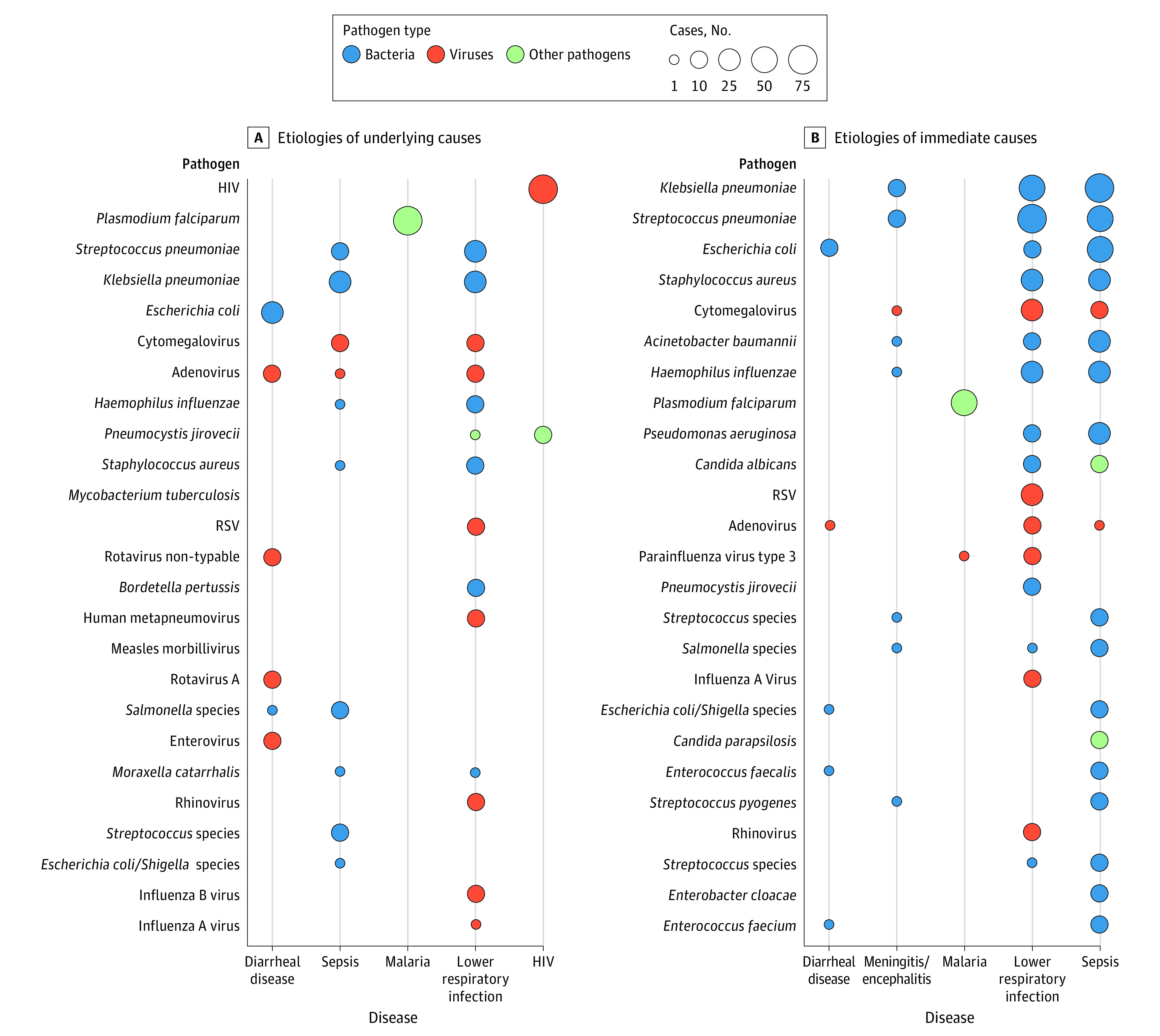
Relative Contribution of Different Pathogens to the Most Common Syndromes or Conditions Common syndromes or conditions are identified as underlying causes (270 disease-pathogen pairs; A) and immediate causes (479 disease-pathogen pairs; B). The size of the bubble is proportional to the number of cases detected (see eTable 7 in Supplement 1 for details). Note that, according to coding practice, deaths with only 1 cause identified have that cause listed as the underlying cause and no immediate cause of death assigned.

Nearly half (306 of 632 [48.4%]) of all deaths had an infectious disease as the underlying COD, but an infection was present somewhere in the causal chain in 549 of 632 deaths (86.9%). HIV infection (75 deaths [11.9%]) was the most common infectious condition determined to be the underlying COD and the fourth most common overall cause when also considering intermediate and immediate CODs. Of all 632 deaths, 29 (4.6%) had sepsis as the underlying COD. *Klebsiella pneumoniae* (155 [28.2%] of all infectious deaths, 127 [81.9%] of which were considered nosocomial), *Plasmodium falciparum* (122 [22.2%]), and *Streptococcus pneumoniae* (109 [19.9%]) were the 3 pathogens most frequently determined to have caused deaths when considering any position in the causal chain (Table 2). Importantly, cytomegalovirus (57 [10.4%] of all infectious deaths) and *Acinetobacter baumannii* (39 [7.1%]; 35 of 39 [89.7%] considered nosocomial) were responsible for substantial mortality in our series. Among underlying CODs (Figure 3A; eTable 7 in Supplement 1), HIV, *P falciparum*, and *S pneumoniae* (as a cause of LRTI and sepsis) were important precipitating factors for the clinical episode resulting in death, whereas among immediate CODs, gram-negative (*K pneumoniae* or *Escherichia coli*) and positive bacteria (*S pneumoniae* and *Staphylococcus aureus*) were involved in the LRTI or sepsis cases that ended up causing death (Figure 3B; eTable 7 in Supplement 1). Of 549 deaths from an infectious disease, 283 (51.5%) had more than 1 pathogen identified in the causal chain, 212 (42.8%) had 1 pathogen identified, and 54 (9.8%) did not have a specific pathogen identified. Most bacteria and viruses were typically detected simultaneously with other infections (eTable 8 in Supplement 1).

**Table 2.** Pathogens Most Frequently Determined to Have Caused Deaths According to Their Position in the Causal Chain Leading to Death for All Deaths and Deaths With at Least 1 Infection in the Causal Chain

Pathogen	No. of pathogens	Pathogens among deaths of any cause, % (n = 632)^a^	Pathogens among infectious deaths, % (n = 549)^b^
Underlying cause			
HIV	75	11.9	13.7
* Plasmodium falciparum*	68	10.8	12.4
* Streptococcus pneumoniae*	26	4.1	4.7
* Klebsiella pneumoniae*	23	3.6	4.2
Cytomegalovirus	13	2.1	2.4
* Escherichia coli*	12	1.9	2.2
Adenovirus	10	1.6	1.8
*Haemophilus influenzae*^c^	9	1.4	1.6
Rotavirus	8	1.3	1.5
* Pneumocystis jirovecii*	5	0.8	0.9
Immediate cause			
* Klebsiella pneumoniae*	113	17.9	20.6
* Streptococcus pneumoniae*	83	13.1	15.1
* Plasmodium falciparum*	43	6.8	7.8
* Escherichia coli*	39	6.2	7.1
Cytomegalovirus	32	5.1	5.8
* Staphylococcus aureus*	31	4.9	5.5
*Haemophilus influenzae*^d^	30	4.7	5.5
* Acinetobacter baumannii*	21	3.3	3.8
* Pseudomonas aeruginosa*	21	3.3	3.8
Adenovirus	13	2.1	2.4
Any cause			
* Klebsiella pneumoniae*	155	24.5	28.2
* Plasmodium falciparum*	122	19.3	22.2
* Streptococcus pneumoniae*	109	17.2	19.9
HIV	75	11.9	13.7
* Escherichia coli*	58	9.2	10.6
Cytomegalovirus	57	9.0	10.4
*Haemophilus influenzae*^e^	46	7.3	8.4
* Staphylococcus aureus*	43	6.8	7.8
* Acinetobacter baumannii*	39	6.2	7.1
Adenovirus	27	4.3	4.9

^a^ Total percentage was calculated as the number of counts divided by the total number of deaths (N = 632).

^b^ Relative percentage was calculated as the number of counts divided by deaths having an underlying, immediate, or any cause of infectious origin (n = 549).

^c^ Includes 1 type A, 1 type B, and 7 untyped.

^d^ Includes 2 type A, 2 type B, and 26 untyped.

^e^ Includes 3 type A, 2 type B, and 41 untyped.

### Preventable Deaths and Opportunities for Intervention

Overall, the DeCoDe panelists considered 494 of 632 deaths (78.2%) preventable and 26 of 632 deaths (4.1%) preventable under certain conditions (eTable 9 in Supplement 1). The proportion of preventable deaths did not differ significantly between infants and children or by location of death (community vs facility). According to the DeCoDe panelists, improvements in clinical management could have prevented 296 of 632 deaths (46.8%), although improved health-seeking behavior and access to health care (160 of 632 [25.3%]), more health education at the community level (147 of 632 [23.3%]), and enhanced nutritional support (134 of 632 [21.2%]) were also commonly noted prevention measures (eFigure 3 in Supplement 1).

## Discussion

We have presented highly granular data on the causal chains and underlying causes of 632 postneonatal deaths, derived from 4 years of cumulative mortality surveillance in 7 sites from the CHAMPS Network. This analysis offers insight into the complexities and interrelations behind each of these deaths. Only one-quarter of enrolled deaths presented a single COD. Previous work^9^ has characterized the value of considering multiple causes of death and highlighted the importance of considering the chain of events leading to death (rather than the underlying cause alone) to better guide research and prevention priorities aimed at reducing child deaths.

Malnutrition was the most common underlying COD, initiating the cascade of events in 16.5% of the deaths, but panelists considered it a relevant problem in nearly a quarter (23.7%) of all fatal cases, highlighting the prominence of this condition in the different study sites. Notably, even if 45.5% of all studied deaths met criteria for severe underweight, DeCoDe panelists only attributed causality following detailed diagnostic standards,^21^ and its mere presence was often insufficient to consider it a COD. Our results contrast with the World Health Organization and other modeling group estimates, which do not typically consider malnutrition as an underlying COD.^1^ The COD descriptions should capture the significance of malnutrition, given its clear predisposing role to infectious deaths, and should evolve from the traditional 2-dimensional “one death one cause” dogma, epitomized by the typical pie chart distribution of COD often used^1,22^; embracing more complex causal chains is necessary to more accurately identify needed interventions.

Heavily influenced by deaths enrolled in the South Africa, Mozambique, and Kenya sites, HIV/AIDS was the second most common underlying COD (11.9%) among enrolled deceased participants. Notably, these deaths are happening despite mother-to-child transmission having markedly decreased in those countries. Even if HIV-infected infants now constitute a clear minority of the birth cohort, they still contribute disproportionately to deaths in children younger than 5 years, highlighting the importance of HIV prevention strategies to improve child survival. The unavailability of individual viral load data hinders a more detailed interpretation of those cases. Lower respiratory tract infections, already well characterized as the number 1 single killer of children,^23^ were only the fifth most common underlying COD in frequency; however, LRTIs were the most common overall cause of death, present in the causal chain in nearly half of all deaths. Similarly, sepsis, relatively infrequent as an underlying COD (4.6%), was detected in the causal chain of 39.4% of the cases. Malaria (present in 19.5% of deaths, despite being absent from sites such as South Africa, Ethiopia, or Bangladesh) and diarrheal diseases (14.2% of the cases) were other common causes of death along with congenital birth defects (mostly as an underlying COD). Importantly, conditions affecting women during pregnancy (maternal factors) also played a major role in 7.9% of the postneonatal deaths, although less commonly than for deaths occurring in the neonatal period,^24^ even though our estimates may be biased by the inconsistent availability of maternal data for deaths, particularly for older children. Taken together, our findings confirm the major CODs among infants and childhood previously described in the literature^1,22^ but provide new estimates of their interrelations, relative importance at each site, and role using data we believe are more accurate.

Most CHAMPS sentinel sites are in settings where, epidemiologically, there is a double burden of communicable and noncommunicable diseases. Our data demonstrate the disproportionate contribution of infections to mortality, with pathogenic microorganisms determined by DeCoDe panels to be in the causal chain of nearly 8 of every 10 deaths. Although the MITS method may be biased toward overdetection of infections^25^ and underperform in the detection of noncommunicable conditions,^26^ single and mixed infections are clearly major contributors to mortality in the postneonatal period. The significant mortality still caused by *S pneumoniae* and *H influenzae* indicates gaps in protection against severe disease on account of vaccination coverage, disease caused by nonvaccine serotypes, or the high prevalence of underlying conditions reducing vaccine effectiveness, such as malnutrition.

Our data also highlight the importance of other pathogens that are not typically identified as major causes of childhood mortality. Notably, *K pneumoniae* (155 of 549 deaths [28.2%] from infectious diseases or 155 of 632 [24.5% ] of all deaths) was by far the most common pathogen detected. Although identification of this gram-negative bacterium in postmortem samples can suggest postmortem overgrowth and contamination, DeCoDe panelists attributed causality to this organism only if certain of its pathophysiologic involvement in the fatal event, for instance when confirming its presence associated with tissue lesions through immunohistochemical analysis. *Klebsiella pneumoniae* was uncommonly attributed in the etiology of childhood pneumonia in the pivotal PERCH (Pneumonia Etiology Research for Child Health) case-control study,^27^ which primarily included children who survived their pneumonia episodes and where scarce lung samples were available. Our findings warrant a more detailed investigation of its overall contribution to child deaths, likely much larger than previously considered. Similarly, the etiologic fraction of cytomegalovirus found in PERCH, identified in a comparable number of cases and controls, did not pinpoint cytomegalovirus as an important cause of pneumonia. Our data confirm cytomegalovirus as an important contributor to local and systemic disease and death and not exclusively in association with HIV exposure or confirmed infection. Of note, the distribution of infectious origins identified in our series of deaths is expected to differ from etiologic studies of living children, in part because antibiotic therapy would selectively prevent deaths from certain pathogens and in part because our sampling and testing are more extensive than is possible in studies of living children.

A project such as CHAMPS that focuses on in-depth analysis of the causes of individual deaths is only useful if it offers solutions to improve child survival. In this respect, DeCoDe panelists were requested to reflect on the preventability of each death and propose how these deaths could have been averted. Reassuringly, up to 82.3% of the deaths were deemed preventable or potentially preventable, opening many opportunities for targeted interventions and predictable impact. Many of the areas where improvements are needed reflect systemic and deep-rooted problems and broad categories of issues that cannot be easily solved without significant investments, which can be prioritized according to the insights and relative weight observed in our data. In addition, more details are needed to better direct investments in improving the quality of programs, such as what types of failures of clinical management led to many of the deaths. By also including deaths in the community, CHAMPS data provide specific descriptions of what needs to be done to prevent those deaths that are often invisible to the health care system.

### Limitations

Important limitations of this analysis relate to issues regarding representativeness of the site-specific data. Despite Bangladesh being one of the CHAMPS sites, this article essentially presents data on postneonatal deaths from sub-Saharan Africa, and their relevance for global estimates therefore is limited to this continent. New CHAMPS-like COD data from other high-mortality settings in Asia are needed. Additionally, within the African sites, the distributions of the DeCoDe MITS data are heavily skewed toward South Africa and Western Kenya (both remarkable for being HIV hotspots). Interpretation of the data should therefore be specific to the sites sampled, and the patterns of causes should be considered potentially biased by the differences in site-to-site epidemiology and sampling weights.

The CHAMPS DeCoDe method used for COD attribution is also not free from its own limitations, including its challenging standardization across sites, the inherent challenges of establishing the time ordering of events, and its dependence on experts (and their subjectivities) from each site, potentially leading to significant site-to-site variability. Additionally, CHAMPS data do not account for the socioeconomic and structural determinants that influence mortality, likely influencing mortality among children younger than 5 years in LMICs.

Finally, we were unable to quantify the potential contribution of the newly emerged SARS-CoV-2 virus among our cases, given that samples were screened in real time on sites using the predesigned TaqMan Array cards, which lacked this pathogen. We are currently investigating the prevalence of SARS-CoV-2 by testing stored samples, although we do not expect that SARS-CoV-2 contributed significantly to CHAMPS deaths, given our current knowledge on pediatric COVID-19 and how the pandemic has affected sub-Saharan African countries.^28^

## Conclusions

Despite these limitations, the CHAMPS results reported in this cross-sectional study provide carefully generated COD information that can improve estimates of the causes of childhood mortality. Ongoing efforts to strengthen vital registration systems have improved the capacity to account for mortality,^29^ but attributing a credible cause to each of those deaths often remains an unsurmountable challenge. The significant variability in COD estimates according to the different models used^30^ hints at the imperfection of the models and the need for a collaborative approach in rethinking the science behind them. The significantly improved and open access COD source data derived from CHAMPS efforts in specific high-mortality LMIC settings can contribute to redefining the gold standard approach for constructing COD estimates.

## Notes

Supplement 1. **eFigure 1.** Geographical Distribution and Number of Post-Neonatal Deaths Recruited Within the Different CHAMPS Network Sites **eFigure 2.** Sunburst Diagram Allowing the Navigation Across Individual Causal Pathways (Full Sequence of Events) for Each of the 632 Included Deaths in This Analysis **eFigure 3.** Opportunities for Intervention Identified by Expert DeCoDe Panelists Which Could Have Prevented Some of the Deaths **eTable 1.** Median Number of Causes in the Chain of Events Leading to Death for the 5 Most Commonly Occurring Underlying Causes of Death and the Most Frequent Secondary Events Associated With the Underlying Diagnosis **eTable 2.** Number of Identified Conditions in Causal Chain Leading to Death, by Selected Case Characteristics, CHAMPS Network (Sites in Manhiça, Mozambique; Soweto, South Africa; Kisumu and Siaya County, Kenya; Bamako, Mali; Baliakandi, Bangladesh; Makeni, Sierra Leone; Harar and Kersa, Ethiopia), December 2016–December 2020 **eTable 3.** Median Number of Identified Conditions (Range) in Causal Chain Leading to Death by Age Group and CHAMPS Site **eTable 4.** Median Number of Different Causes in Chain of Events Leading to Death for 5 Most Common Underlying Causes and Most Frequently Associated Events **eTable 5.** Underlying Data for the Sankey Diagram in Figure 1 **eTable 6.** Frequencies of Causes of Death According to Place of Death (Community vs. Facility) and to Position in the Causal Chain (Underlying, Immediate, or All Causes Pooled Together) (Sites in Manhiça, Mozambique; Soweto, South Africa; Kisumu and Siaya County, Kenya; Bamako, Mali; Baliakandi, Bangladesh; Makeni, Sierra Leone; Harar and Kersa, Ethiopia), December 2016–December 2020 **eTable 7.** Disease Events Caused by a Given Pathogen **eTable 8.** Number of Times Pathogens Were Determined to Be in the Causal Chain Leading to Death **eTable 9.** Preventability of Studied Deaths According to the Decode Panel, Stratified by Age Groups and Place of Death

Supplement 2.
Nonauthor Collaborators

Supplement 3.
Data Sharing Statement
