# Supplementary material for: Causes of Death Among Infants and Children in the Child Health and Mortality Prevention Surveillance (CHAMPS) Network
Source: JAMA Netw Open. 2023 Jul 26;6(7):e2322494. doi: 10.1001/jamanetworkopen.2023.22494 (PMC10372710; doi:10.1001/jamanetworkopen.2023.22494)
Supplement: Supplement 1. — eFigure 1. Geographical Distribution and Number of Post-Neonatal Deaths Recruited Within the Different CHAMPS Network Sites eFigure 2. Sunburst Diagram Allowing the Navigation Across Individual Causal Pathways (Full Sequence of Events) for Each of the 632 Included Deaths in This Analysis eFigure 3. Opportunities for Intervention Identified by Expert DeCoDe Panelists Which Could Have Prevented Some of the Deaths eTable 1. Median Number of Causes in the Chain of Events Leading to Death for the 5 Most Commonly Occurring Underlying Causes of Death and the Most Frequent Secondary Events Associated With the Underlying Diagnosis eTable 2. Number of Identified Conditions in Causal Chain Leading to Death, by Selected Case Characteristics, CHAMPS Network (Sites in Manhiça, Mozambique; Soweto, South Africa; Kisumu and Siaya County, Kenya; Bamako, Mali; Baliakandi, Bangladesh; Makeni, Sierra Leone; Harar and Kersa, Ethiopia), December 2016–December 2020 eTable 3. Median Number of Identified Conditions (Range) in Causal Chain Leading to Death by Age Group and CHAMPS Site eTable 4. Median Number of Different Causes in Chain of Events Leading to Death for 5 Most Common Underlying Causes and Most Frequently Associated Events eTable 5. Underlying Data for the Sankey Diagram in Figure 1 eTable 6. Frequencies of Causes of Death According to Place of Death (Community vs. Facility) and to Position in the Causal Chain (Underlying, Immediate, or All Causes Pooled Together) (Sites in Manhiça, Mozambique; Soweto, South Africa; Kisumu and Siaya County, Kenya; Bamako, Mali; Baliakandi, Bangladesh; Makeni, Sierra Leone; Harar and Kersa, Ethiopia), December 2016–December 2020 eTable 7. Disease Events Caused by a Given Pathogen eTable 8. Number of Times Pathogens Were Determined to Be in the Causal Chain Leading to Death eTable 9. Preventability of Studied Deaths According to the Decode Panel, Stratified by Age Groups and Place of Death [file jamanetwopen-e2322494-s001.pdf]

Supplementary Online Content

Bassat Q, Blau DM, Ogbuanu IU, et al; Child Health and Mortality Prevention Surveillance (CHAMPS) Network. Causes of death among infants and children in the Child Health and Mortality Prevention Surveillance (CHAMPS) Network. *JAMA Netw Open*. 2023;6(7):e2322494. doi:10.1001/jamanetworkopen.2023.22494

eFigure 1. Geographical Distribution and Number of Post-Neonatal Deaths Recruited Within the Different CHAMPS Network Sites

eFigure 2. Sunburst Diagram Allowing the Navigation Across Individual Causal Pathways (Full Sequence of Events) for Each of the 632 Included Deaths in This Analysis

eFigure 3. Opportunities for Intervention Identified by Expert DeCoDe Panelists Which Could Have Prevented Some of the Deaths

eTable 1. Median Number of Causes in the Chain of Events Leading to Death for the 5 Most Commonly Occurring Underlying Causes of Death and the Most Frequent Secondary Events Associated With the Underlying Diagnosis

eTable 2. Number of Identified Conditions in Causal Chain Leading to Death, by Selected Case Characteristics, CHAMPS Network (Sites in Manhiça, Mozambique; Soweto, South Africa; Kisumu and Siaya County, Kenya; Bamako, Mali; Baliakandi, Bangladesh; Makeni, Sierra Leone; Harar and Kersa, Ethiopia), December 2016–December 2020

eTable 3. Median Number of Identified Conditions (Range) in Causal Chain Leading to Death by Age Group and CHAMPS Site

eTable 4. Median Number of Different Causes in Chain of Events Leading to Death for 5 Most Common Underlying Causes and Most Frequently Associated Events

eTable 5. Underlying Data for the Sankey Diagram in Figure 1

eTable 6. Frequencies of Causes of Death According to Place of Death (Community vs. Facility) and to Position in the Causal Chain (Underlying, Immediate, or All Causes Pooled Together) (Sites in Manhiça, Mozambique; Soweto, South Africa; Kisumu and Siaya County, Kenya; Bamako, Mali; Baliakandi, Bangladesh; Makeni, Sierra Leone; Harar and Kersa, Ethiopia), December 2016–December 2020

eTable 7. Disease Events Caused by a Given Pathogen

eTable 8. Number of Times Pathogens Were Determined to Be in the Causal Chain Leading to Death

eTable 9. Preventability of Studied Deaths According to the Decode Panel, Stratified by Age Groups and Place of Death

This supplementary material has been provided by the authors to give readers additional information about their work.

© 2023 Bassat Q et al. *JAMA Network Open.*

eFigure 1: Geographical distribution and number of post-neonatal deaths recruited within the different CHAMPS network sites.

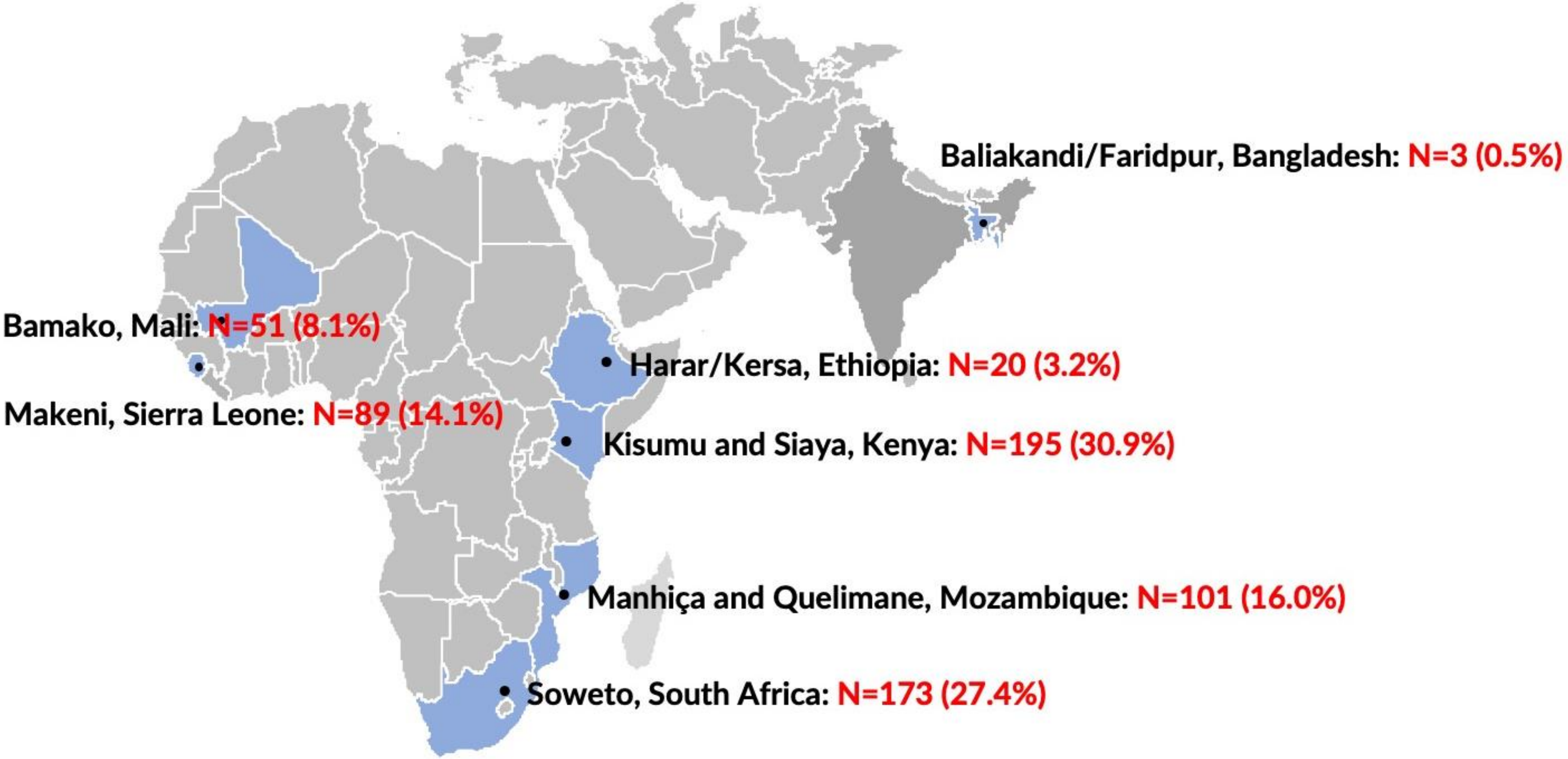

© 2023 Bassat Q et al. *JAMA Network Open.*

eFigure 2: Sunburst diagram allowing the navigation across individual causal pathways (full sequence of events) for each of the 632 included deaths in this analysis. To access this interactive material, please use the following link: https://champs-sunburst.netlify.app

Explanation: By placing the cursor in the middle circle (n=632, 100% of the cases), and navigating outwards by radian, one can see how many cases 8and what proportion) have been seen for each combination of diagnoses as part of the causal chain. As an example, we are showing the screenshots of the radian:

Total (100%)>>
+Malnutrition (16.5%) >>
+Lower respiratory infections (4.43%)>>
+Sepsis (1.74%)>>
+Meningitis/Encephalitis (0.158%) >>
+Diarrheal diseases (0.158%)

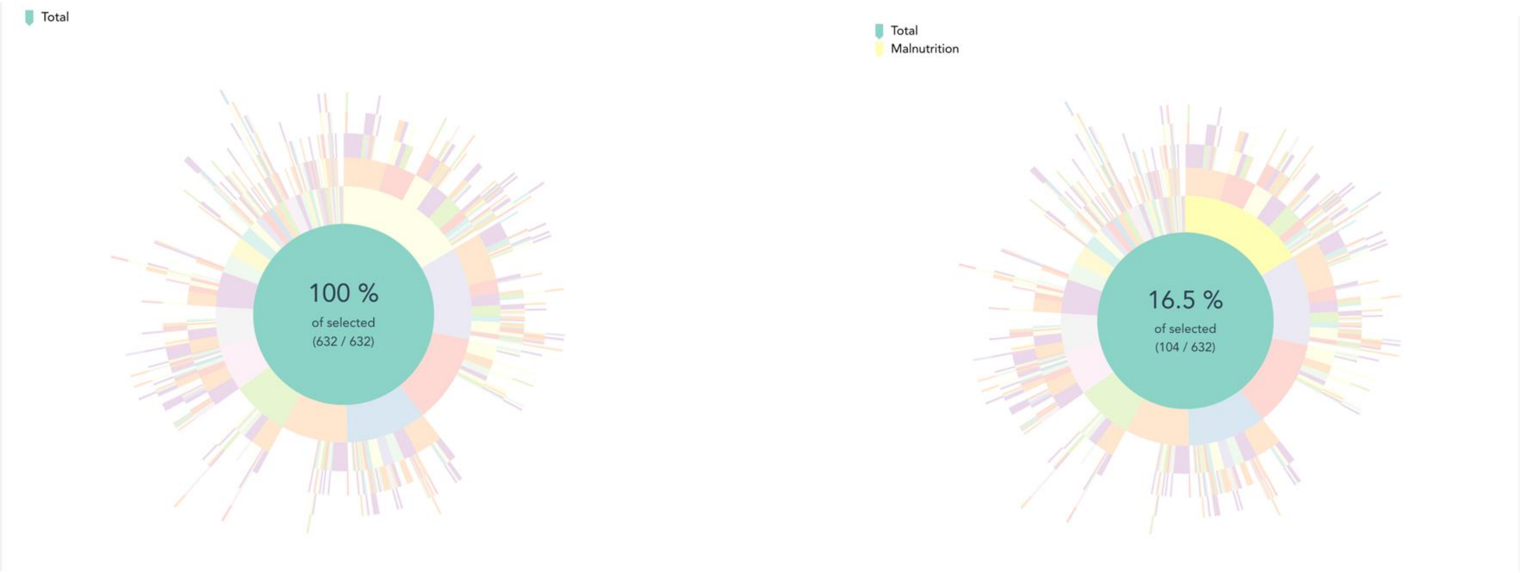

© 2023 Bassat Q et al. *JAMA Network Open.*

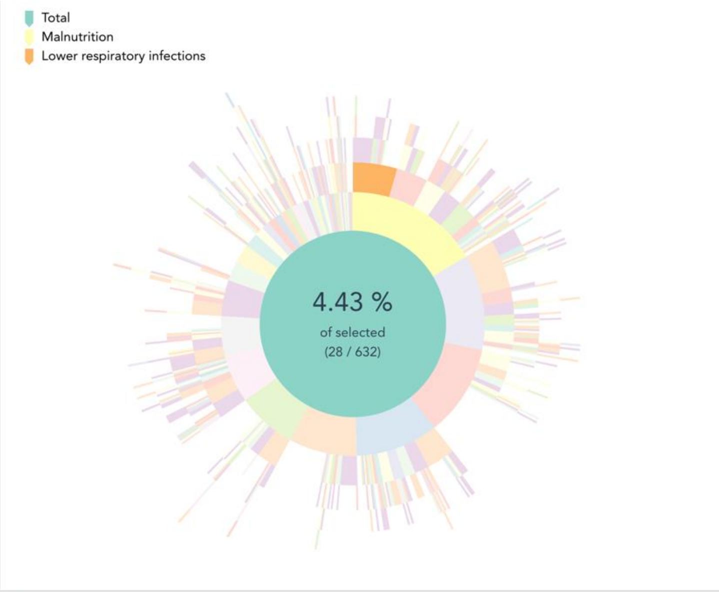

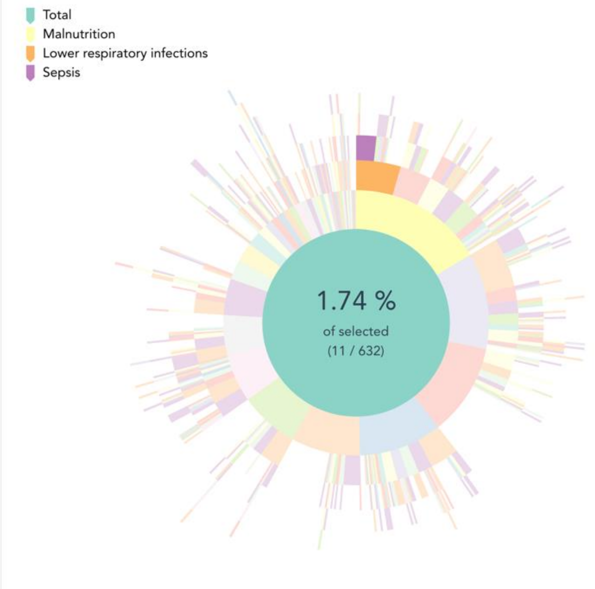

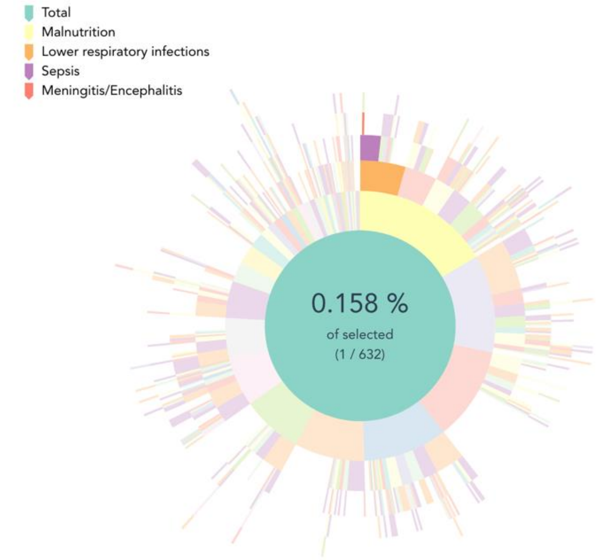

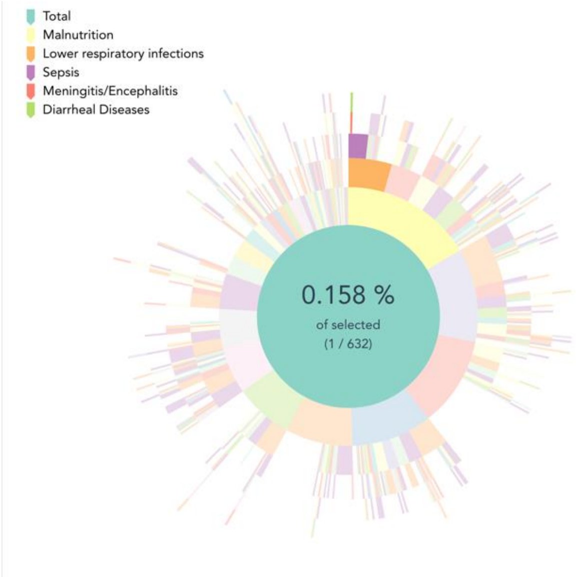

© 2023 Bassat Q et al. *JAMA Network Open.*

eFigure 3: Opportunities for intervention identified by Expert DeCoDe panelists which could have prevented some of the deaths.

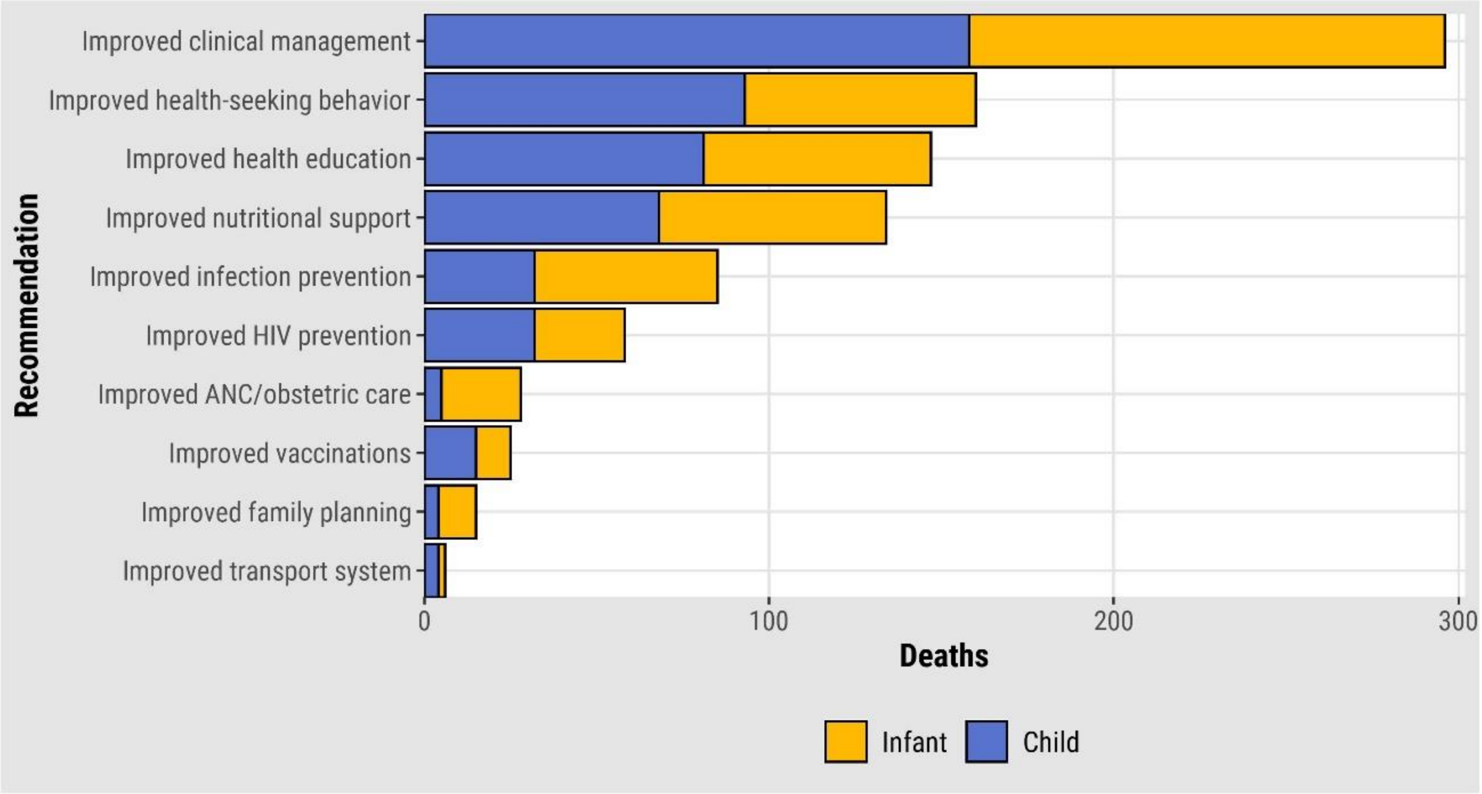

© 2023 Bassat Q et al. *JAMA Network Open.*

eTable 1: Median number of causes in the chain of events leading to death for the 5 most commonly occurring underlying causes of death and the most frequent secondary events associated with the underlying diagnosis.

**Underlying causes**	Counts	Median number events (IQR)	Only 1 Additional cause n (%)	2 additional causes n (%)	3 additional causes n (%)	4 additional causes n (%)	>4 additional causes n (%)	Most commonly reported associated event	Counts of associated most frequent event
Malnutrition	104	3 (2-4)	32 (30.8)	34 (32.7)	27 (26.0)	8 (7.7)	1 (1.0)	Sepsis	56
Human immunodeficiency virus	75	3 (2-3)	25 (33.3)	30 (40.0)	10 (13.3)	7 (9.3)	0 (0)	LRTI	45
Malaria	71	1 (1-2)	17 (23.9)	8 (11.3)	5 (7.0)	0 (0)	0 (0)	Anemias	16
Congenital birth defects	64	3 (2-3)	22 (34.4)	24 (37.5)	14 (21.9)	1 (1.6)	0 (0)	LRTI	34
Lower respiratory tract infection	53	1 (1-2)	16 (30.2)	5 (9.4)	1 (1.9)	1 (1.9)	0 (0)	Sepsis	16

© 2023 Bassat Q et al. *JAMA Network Open.*

eTable 2: Number of identified conditions in causal chain leading to death, by selected case characteristics, CHAMPS Network (sites in Manhiça, Mozambique; Soweto, South Africa; Kisumu and Siaya county, Kenya; Bamako, Mali; Baliakandi, Bangladesh; Makeni, Sierra Leone; Harar and Kersa, Ethiopia), December 2016–December 2020.

	0 (N=15)	1 (N=156)	2 (N=194)	3 (N=146)	4 (N=86)	5 (N=29)	6 (N=4)	7 (N=2)	Total (N=632)
**Age group**									
Infant (28 days to <12 months)	11 (73.3)	74 (47.4)	107 (55.2)	80 (54.8)	42 (48.8)	14 (48.3)	2 (50.0)	1 (50.0)	331 (52.4)
Child (12 months to <60 Months)	4 (26.7)	82 (52.6)	87 (44.8)	66 (45.2)	44 (51.2)	15 (51.7)	2 (50.0)	1 (50.0)	301 (47.6)
**CHAMPS Site**									
South Africa	1 (6.7)	30 (19.2)	50 (25.8)	47 (32.2)	30 (34.9)	12 (41.4)	2 (50.0)	1 (50.0)	173 (27.4)
Mozambique	1 (6.7)	34 (21.8)	42 (21.6)	17 (11.6)	7 (8.1)	0 (0)	0 (0)	0 (0)	101 (16.0)
Kenya	6 (40.0)	67 (42.9)	72 (37.1)	34 (23.3)	15 (17.4)	1 (3.4)	0 (0)	0 (0)	195 (30.8)
Ethiopia	0 (0)	1 (0.6)	2 (1.0)	6 (4.1)	4 (4.7)	6 (20.7)	1 (25.0)	0 (0)	20 (3.2)
Sierra Leone	6 (40.0)	12 (7.7)	17 (8.8)	25 (17.1)	24 (27.9)	4 (13.8)	1 (25.0)	0 (0)	89 (14.1)
Mali	1 (6.7)	12 (7.7)	10 (5.2)	16 (11.0)	5 (5.8)	6 (20.7)	0 (0)	1 (50.0)	51 (8.1)
Bangladesh	0 (0)	0 (0)	1 (0.5)	1 (0.5)	1 (0.5)	0 (0)	0 (0)	0 (0)	3 (0.5)
**Location and timing of death**									
Facility, ≤24 hours after admission	4 (26.7)	64 (41.0)	65 (33.5)	43 (29.5)	28 (32.6)	8 (27.6)	0 (0)	0 (0)	212 (33.5)
Facility, >24 hours after admission	1 (6.7)	34 (21.8)	68 (35.1)	76 (52.1)	45 (52.3)	16 (55.2)	3 (75.0)	1 (50.0)	244 (38.6)
Community	10 (66.7)	58 (37.2)	61 (31.4)	27 (18.5)	13 (15.1)	5 (17.2)	1 (25.0)	1 (50.0)	176 (27.8)

eTable 3: Median number of identified conditions (range) in causal chain leading to death by age group ad CHAMPS site

	South Africa (N=173)	Mozambique (N=101)	Kenya (N=195)	Ethiopia (N=20)	Sierra Leone (N=89)	Mali (N=51)	Bangladesh (N=3)	Total (N=632)
**Age group**								
Infant (28 days to <12 months)	3 (1-7)	2 (0-3)	2 (0-5)	3 (1-4)	2.5 (0-6)	3 (0-5)	2.5 (2-3)	2 (0-7)
Child (12 months to <60 Months)	2 (0-6)	2 (1-4)	2 (0-4)	5 (2-6)	3 (0-5)	3 (1-7)	4 (4-4)	2 (0-7)

© 2023 Bassat Q et al. *JAMA Network Open.*

eTable 4: Median number of different causes in chain of events leading to death for 5 most common underlying causes and most frequently associated events

	Counts	Median length of chain of events	Most frequently associated cause		Second most frequently associated cause		Third most frequently associated cause	
			**Cause**	**Number**	**Cause**	**Number**	**Cause**	**Number**
Malnutrition	104	3.0	Sepsis	56	Lower respiratory infections	52	Malaria	28
HIV	75	3.0	Lower respiratory infections	45	Sepsis	31	Malaria	13
Malaria	71	1.0	Anemias	16	Other respiratory disease	9	Sepsis	7
Congenital birth defects	64	3.0	Lower respiratory infections	34	Sepsis	27	Other respiratory disease	10
Lower Respiratory infections	53	1.0	Sepsis	16	Meningitis/Encephalitis	3	Other respiratory disease	3

© 2023 Bassat Q et al. *JAMA Network Open.*

eTable 5. Underlying data for the Sankey diagram in **Figure 1**. Cases from Table Supplementary table S5 are discretized (i.e. rounded to the ceiling integer).

**From_Stage**	**From**	**To_Stage**	**To**	**Cases**
Intermediate	Lower respiratory infections	Immediate	Sepsis	55
Underlying	Malnutrition	Intermediate	Lower respiratory infections	19
Underlying	HIV	Intermediate	Lower respiratory infections	16
Underlying	Malnutrition	Intermediate	Anemias	15
Intermediate	Sepsis	Immediate	Lower respiratory infections	15
Underlying	HIV	Immediate	Lower respiratory infections	13
Intermediate	Meningitis/Encephalitis	Immediate	Sepsis	12
Intermediate	Malnutrition	Immediate	Sepsis	11
Underlying	Malnutrition	Intermediate	Malaria	11
Underlying	Lower respiratory infections	Immediate	Sepsis	10
Underlying	Congenital birth defects	Immediate	Lower respiratory infections	10
Intermediate	Malaria	Immediate	Sepsis	10
Intermediate	Diarrheal Diseases	Immediate	Sepsis	10
Underlying	Malnutrition	Immediate	Lower respiratory infections	9
Intermediate	Anemias	Immediate	Sepsis	9
Underlying	Malnutrition	Immediate	Malaria	8
Underlying	Diarrheal Diseases	Immediate	Lower respiratory infections	8
Underlying	Sepsis	Immediate	Lower respiratory infections	8
Intermediate	Malnutrition	Immediate	Lower respiratory infections	8
Underlying	Malnutrition	Intermediate	Diarrheal Diseases	8
Underlying	Congenital birth defects	Intermediate	Malnutrition	8
Underlying	Congenital birth defects	Intermediate	Lower respiratory infections	8
Underlying	Malnutrition	Immediate	Sepsis	7
Underlying	Congenital birth defects	Intermediate	Other	7
Underlying	HIV	Intermediate	Malnutrition	7

© 2023 Bassat Q et al. *JAMA Network Open.*

Underlying	Malnutrition	Intermediate	Sepsis	7
Intermediate	Other respiratory disease	Immediate	Lower respiratory infections	7
Underlying	Neonatal preterm birth	Intermediate	Lower respiratory infections	7
Underlying	Injury	Intermediate	Lower respiratory infections	7
Underlying	Malaria	Immediate	Anemias	6
Underlying	HIV	Immediate	Malaria	6
Underlying	Malnutrition	Immediate	Diarrheal Diseases	6
Intermediate	Other	Immediate	Sepsis	6
Underlying	HIV	Intermediate	Diarrheal Diseases	6
Underlying	HIV	Intermediate	Sepsis	6
Underlying	Injury	Immediate	Sepsis	5
Intermediate	Anemias	Immediate	Malaria	5
Underlying	Malaria	Immediate	Lower respiratory infections	5
Underlying	Diarrheal Diseases	Immediate	Sepsis	5
Underlying	HIV	Intermediate	Other infections	5
Intermediate	Malaria	Immediate	Lower respiratory infections	5
Underlying	Malaria	Intermediate	Anemias	5
Intermediate	Anemias	Immediate	Lower respiratory infections	5
Underlying	Neonatal preterm birth	Intermediate	Sepsis	5
Intermediate	Other infections	Immediate	Sepsis	5
Underlying	Malnutrition	Intermediate	Meningitis/Encephalitis	5
Intermediate	Other infections	Immediate	Lower respiratory infections	5
Intermediate	Lower respiratory infections	Immediate	Other infections	4
Intermediate	Malnutrition	Immediate	Malaria	4
Underlying	Neonatal preterm birth	Immediate	Lower respiratory infections	4
Underlying	Congenital birth defects	Intermediate	Other respiratory disease	4
Underlying	Neonatal preterm birth	Immediate	Sepsis	4
Underlying	Sepsis	Immediate	Meningitis/Encephalitis	4
Underlying	Neonatal preterm birth	Intermediate	Meningitis/Encephalitis	4
Intermediate	Other respiratory disease	Immediate	Sepsis	4

© 2023 Bassat Q et al. *JAMA Network Open.*

Underlying	Diarrheal Diseases	Intermediate	Lower respiratory infections	4
Underlying	HIV	Intermediate	Malaria	4
Intermediate	Meningitis/Encephalitis	Immediate	Lower respiratory infections	4
Underlying	Malaria	Intermediate	Malnutrition	4
Underlying	Congenital birth defects	Intermediate	Sepsis	4
Underlying	Neonatal preterm birth	Intermediate	Other neonatal disorders	4
Intermediate	Other neurological disorders	Immediate	Lower respiratory infections	4
Underlying	Malaria	Immediate	Sepsis	3
Intermediate	Diarrheal Diseases	Immediate	Other respiratory disease	3
Underlying	Malaria	Immediate	Other respiratory disease	3
Underlying	Diarrheal Diseases	Immediate	Other respiratory disease	3
Underlying	HIV	Immediate	Sepsis	3
Underlying	Congenital birth defects	Immediate	Other respiratory disease	3
Underlying	Congenital birth defects	Immediate	Sepsis	3
Underlying	Sepsis	Intermediate	Anemias	3
Intermediate	Diarrheal Diseases	Immediate	Lower respiratory infections	3
Intermediate	Sepsis	Immediate	Other respiratory disease	3
Intermediate	Anemias	Immediate	Other respiratory disease	3
Underlying	Neonatal preterm birth	Intermediate	Malnutrition	3
Underlying	Injury	Intermediate	Malnutrition	3
Underlying	Malaria	Intermediate	Other respiratory disease	3
Intermediate	Sepsis	Immediate	Other infections	3
Intermediate	Malnutrition	Immediate	Anemias	3
Intermediate	Malaria	Immediate	Diarrheal Diseases	3
Underlying	Injury	Intermediate	Sepsis	3
Underlying	Congenital birth defects	Intermediate	Neonatal preterm birth	3
Underlying	HIV	Intermediate	Anemias	3
Underlying	Lower respiratory infections	Immediate	Other respiratory disease	2
Underlying	Other neonatal disorders	Immediate	Lower respiratory infections	2
Underlying	Other skin and subcutaneous	Immediate	Sepsis	2

© 2023 Bassat Q et al. *JAMA Network Open.*

Underlying	Paralytic ileus and intestinal	Immediate	Other respiratory disease	2
Underlying	Other	Immediate	Sepsis	2
Underlying	Other respiratory disease	Immediate	Lower respiratory infections	2
Underlying	Congenital birth defects	Intermediate	Other infections	2
Intermediate	Malnutrition	Immediate	Other respiratory disease	2
Underlying	Sepsis	Immediate	Anemias	2
Underlying	Other neurological disorders	Immediate	Other respiratory disease	2
Underlying	Malnutrition	Intermediate	Other respiratory disease	2
Underlying	Malnutrition	Immediate	Other infections	2
Intermediate	Sepsis	Immediate	Diarrheal Diseases	2
Intermediate	Anemias	Immediate	Meningitis/Encephalitis	2
Intermediate	Lower respiratory infections	Immediate	Meningitis/Encephalitis	2
Intermediate	Other neonatal disorders	Immediate	Sepsis	2
Intermediate	Lower respiratory infections	Immediate	Other respiratory disease	2
Intermediate	Neonatal preterm birth	Immediate	Sepsis	2
Underlying	Malnutrition	Intermediate	Other immunodeficiencies	2
Intermediate	Sepsis	Immediate	Meningitis/Encephalitis	2
Intermediate	Sickle cell disorders	Immediate	Lower respiratory infections	2
Intermediate	Other neonatal disorders	Immediate	Meningitis/Encephalitis	2
Underlying	Diarrheal Diseases	Intermediate	Malnutrition	2
Intermediate	Other	Immediate	Lower respiratory infections	2
Underlying	Neonatal preterm birth	Intermediate	Congenital birth defects	2
Intermediate	Anemias	Immediate	Diarrheal Diseases	2
Underlying	Other endocrine, metabolic,	Intermediate	Lower respiratory infections	2
Intermediate	Tuberculosis	Immediate	Lower respiratory infections	2
Underlying	Injury	Intermediate	Meningitis/Encephalitis	2
Underlying	Congenital birth defects	Intermediate	Meningitis/Encephalitis	2
Intermediate	Congenital birth defects	Immediate	Sepsis	2
Underlying	HIV	Intermediate	Meningitis/Encephalitis	2
Underlying	Lower respiratory infections	Intermediate	Malaria	2

© 2023 Bassat Q et al. *JAMA Network Open.*

Underlying	Sepsis	Intermediate	Malnutrition	2
Intermediate	Kidney Disease	Immediate	Lower respiratory infections	2
Underlying	Diarrheal Diseases	Intermediate	Sepsis	2
Intermediate	Lower respiratory infections	Immediate	Diarrheal Diseases	2
Underlying	Other infections	Intermediate	Malnutrition	1
Intermediate	Lower respiratory infections	Immediate	Anemias	1
Underlying	Injury	Immediate	Other respiratory disease	1
Underlying	Lower respiratory infections	Intermediate	Diarrheal Diseases	1
Underlying	Congenital birth defects	Immediate	Meningitis/Encephalitis	1
Underlying	Measles	Immediate	Lower respiratory infections	1
Underlying	Lower respiratory infections	Immediate	Injury	1
Underlying	Sepsis	Immediate	Other respiratory disease	1
Underlying	Congenital birth defects	Immediate	Liver disease	1
Intermediate	Other respiratory disease	Immediate	Neonatal sepsis	1
Underlying	Heart Diseases	Immediate	Liver Disease	1
Underlying	Paralytic ileus and intestinal	Immediate	Poisoning	1
Intermediate	Sepsis	Immediate	Malaria	1
Underlying	Malnutrition	Intermediate	Sickle cell disorders	1
Underlying	Other skin and subcutaneous	Intermediate	Lower respiratory infections	1
Underlying	HIV	Immediate	Other infections	1
Underlying	HIV	Immediate	Diarrheal Diseases	1
Underlying	Sepsis	Intermediate	Malaria	1
Underlying	Lower respiratory infections	Immediate	Diarrheal Diseases	1
Underlying	Heart Diseases	Intermediate	Other neurological disorders	1
Underlying	Neonatal preterm birth	Intermediate	Malaria	1
Intermediate	Malnutrition	Immediate	Diarrheal Diseases	1
Intermediate	Meningitis/Encephalitis	Immediate	Other infections	1
Underlying	Other	Intermediate	Other respiratory disease	1
Underlying	Neonatal preterm birth	Immediate	Other neonatal disorders	1
Underlying	Other infections	Immediate	Sepsis	1

© 2023 Bassat Q et al. *JAMA Network Open.*

Intermediate	Anemias	Immediate	Malnutrition	1
Underlying	Congenital birth defects	Intermediate	Anemias	1
Intermediate	Other respiratory disease	Immediate	Anemias	1
Underlying	Meningitis/Encephalitis	Immediate	Anemias	1
Underlying	Neonatal preterm birth	Intermediate	Other	1
Underlying	Congenital infection	Immediate	Neonatal sepsis	1
Underlying	Cancer	Immediate	Anemias	1
Underlying	Measles	Intermediate	Sepsis	1
Underlying	Congenital birth defects	Immediate	Epilepsy	1
Underlying	Liver disease	Intermediate	Malnutrition	1
Underlying	Congenital infection	Immediate	Sepsis	1
Underlying	Anemias	Intermediate	Lower respiratory infections	1
Intermediate	Other	Immediate	Other neonatal disorders	1
Underlying	Meningitis/Encephalitis	Intermediate	Sepsis	1
Underlying	Malnutrition	Intermediate	Other	1
Underlying	Meningitis/Encephalitis	Intermediate	Anemias	1
Underlying	Congenital birth defects	Immediate	Malnutrition	1
Underlying	HIV exposure	Immediate	Other skin and subcutaneous	1
Underlying	Congenital birth defects	Immediate	Heart Diseases	1
Intermediate	Lower respiratory infections	Immediate	Tuberculosis	1
Underlying	Sickle cell disorders	Immediate	Sepsis	1
Underlying	Lower respiratory infections	Immediate	Other infections	1
Underlying	Meningitis/Encephalitis	Intermediate	Liver disease	1
Underlying	Lower respiratory infections	Intermediate	Sepsis	1
Underlying	Other	Intermediate	Malnutrition	1
Underlying	Diarrheal Diseases	Immediate	Other infections	1
Intermediate	Liver disease	Immediate	Malnutrition	1
Underlying	Liver Disease	Intermediate	Meningitis/Encephalitis	1
Intermediate	Other	Immediate	Heart Diseases	1
Intermediate	Poisoning	Immediate	Diarrheal Diseases	1

© 2023 Bassat Q et al. *JAMA Network Open.*

Underlying	Tuberculosis	Immediate	Lower respiratory infections	1
Underlying	Injury	Intermediate	Other neurological disorders	1
Underlying	Congenital birth defects	Immediate	Liver Disease	1
Underlying	Liver Disease	Intermediate	Other respiratory disease	1
Underlying	Lower respiratory infections	Intermediate	Other respiratory disease	1
Underlying	Other endocrine, metabolic,	Immediate	Meningitis/Encephalitis	1
Underlying	HIV	Intermediate	Poisoning	1
Underlying	Other neurological disorders	Intermediate	Lower respiratory infections	1
Underlying	Poisoning	Immediate	Lower respiratory infections	1
Underlying	Diarrheal Diseases	Immediate	Other disorders of fluid,	1
Underlying	Injury	Immediate	Other infections	1
Underlying	Malaria	Intermediate	Diarrheal Diseases	1
Underlying	Other infections	Intermediate	Tuberculosis	1
Underlying	Neonatal preterm birth	Intermediate	Kidney Disease	1
Underlying	Tuberculosis	Immediate	Other infections	1
Intermediate	Injury	Immediate	Sepsis	1
Underlying	Injury	Immediate	Lower respiratory infections	1
Intermediate	Other immunodeficiencies	Immediate	Other infections	1
Intermediate	Sepsis	Immediate	Congenital infection	1
Underlying	Other endocrine, metabolic,	Immediate	Congenital birth defects	1
Underlying	Cancer	Immediate	Poisoning	1
Underlying	Measles	Immediate	Sepsis	1
Underlying	Motor neuron disease	Immediate	Lower respiratory infections	1
Underlying	Epilepsy	Immediate	Other respiratory disease	1
Underlying	Neonatal preterm birth	Intermediate	Neonatal encephalopathy	1
Underlying	Lower respiratory infections	Immediate	Meningitis/Encephalitis	1
Intermediate	Neonatal encephalopathy	Immediate	Lower respiratory infections	1
Intermediate	Anemias	Immediate	Other endocrine, metabolic,	1
Intermediate	Other skin and subcutaneous	Immediate	Sepsis	1
Underlying	Malnutrition	Intermediate	Other skin and subcutaneous	1

© 2023 Bassat Q et al. *JAMA Network Open.*

Underlying	Lower respiratory infections	Intermediate	Meningitis/Encephalitis	1
Intermediate	Sepsis	Immediate	Liver Disease	1
Underlying	Upper respiratory infections	Immediate	Lower respiratory infections	1
Underlying	Congenital birth defects	Intermediate	Paralytic ileus and intestinal	1
Intermediate	Paralytic ileus and intestinal	Immediate	Sepsis	1
Underlying	Other	Immediate	Malnutrition	1
Intermediate	Neonatal preterm birth	Immediate	Lower respiratory infections	1
Underlying	Neonatal preterm birth	Intermediate	Other infections	1
Underlying	Lower respiratory infections	Intermediate	Malnutrition	1
Underlying	Lower respiratory infections	Intermediate	Anemias	1
Intermediate	Upper respiratory infections	Immediate	Sepsis	1
Intermediate	Other infections	Immediate	Meningitis/Encephalitis	1
Underlying	Malnutrition	Intermediate	Other infections	1
Underlying	Diarrheal Diseases	Intermediate	Other endocrine, metabolic,	1
Underlying	Diarrheal Diseases	Intermediate	Other neurological disorders	1
Intermediate	Other endocrine, metabolic,	Immediate	Lower respiratory infections	1
Underlying	Congenital infection	Intermediate	Lower respiratory infections	1
Intermediate	Congenital birth defects	Immediate	Lower respiratory infections	1
Underlying	Other	Intermediate	Sepsis	1
Intermediate	Sepsis	Immediate	Anemias	1
Underlying	Malnutrition	Intermediate	Injury	1
Intermediate	Congenital birth defects	Immediate	Other respiratory disease	1
Underlying	Congenital birth defects	Intermediate	Heart Diseases	1
Underlying	Diarrheal Diseases	Intermediate	Meningitis/Encephalitis	1
Intermediate	Anemias	Immediate	Tuberculosis	1
Underlying	Other neonatal disorders	Intermediate	Meningitis/Encephalitis	1
Underlying	Other neonatal disorders	Intermediate	Lower respiratory infections	1
Intermediate	Other immunodeficiencies	Immediate	Sepsis	1
Intermediate	Heart Diseases	Immediate	Lower respiratory infections	1
Intermediate	Congenital infection	Immediate	Lower respiratory infections	1

© 2023 Bassat Q et al. *JAMA Network Open.*

Underlying	Other infections	Intermediate	Anemias	1
Underlying	Congenital birth defects	Intermediate	Upper respiratory infections	1
Underlying	Other infections	Intermediate	Sepsis	1
Intermediate	Sepsis	Immediate	Other endocrine, metabolic,	1
Underlying	Other respiratory disease	Intermediate	Other	1
Underlying	Other respiratory disease	Intermediate	Sickle cell disorders	1
Underlying	Malaria	Intermediate	Injury	1
Underlying	Other	Intermediate	Lower respiratory infections	1
Underlying	Injury	Intermediate	Malaria	1
Intermediate	Anemias	Immediate	Poisoning	1
Intermediate	Malnutrition	Immediate	Poisoning	1
Underlying	Neonatal preterm birth	Intermediate	Congenital infection	1
Intermediate	Malaria	Immediate	Anemias	1
Underlying	Other immunodeficiencies	Intermediate	Congenital birth defects	1
Underlying	Other immunodeficiencies	Intermediate	Lower respiratory infections	1
Underlying	Sepsis	Intermediate	Lower respiratory infections	1
Intermediate	Malaria	Immediate	Tuberculosis	1
Intermediate	Lower respiratory infections	Immediate	Neonatal sepsis	1
Intermediate	Meningitis/Encephalitis	Immediate	Neonatal sepsis	1
Underlying	Neonatal preterm birth	Intermediate	Diarrheal Diseases	1
Underlying	Diarrheal Diseases	Intermediate	Anemias	1
Intermediate	Other gastrointestinal disease	Immediate	Sepsis	1
Underlying	Cancer	Intermediate	Lower respiratory infections	1
Underlying	Cancer	Intermediate	Meningitis/Encephalitis	1
Underlying	Perinatal asphyxia/hypoxia	Intermediate	Meningitis/Encephalitis	1
Underlying	Congenital birth defects	Intermediate	Malaria	1
Intermediate	Other respiratory disease	Immediate	Other endocrine, metabolic,	1
Underlying	Congenital birth defects	Intermediate	Other gastrointestinal disease	1
Intermediate	Poisoning	Immediate	Sepsis	1
Intermediate	Other neonatal disorders	Immediate	Other infections	1

© 2023 Bassat Q et al. *JAMA Network Open.*

Underlying	Other respiratory disease	Intermediate	Poisoning	1
Underlying	Malaria	Intermediate	Lower respiratory infections	1
Underlying	Other respiratory disease	Intermediate	Meningitis/Encephalitis	1
Underlying	Malaria	Intermediate	Other endocrine, metabolic,	1
Intermediate	Other endocrine, metabolic,	Immediate	Other respiratory disease	1
Underlying	Perinatal asphyxia/hypoxia	Intermediate	Lower respiratory infections	1
Intermediate	Other	Immediate	Other respiratory disease	1
Intermediate	Other skin and subcutaneous	Immediate	Lower respiratory infections	1
Underlying	HIV	Intermediate	Other skin and subcutaneous	1
Intermediate	Diarrheal Diseases	Immediate	Anemias	1
Underlying	Diarrheal Diseases	Intermediate	Other disorders of fluid,	1
Underlying	Diarrheal Diseases	Intermediate	Kidney Disease	1
Intermediate	Malnutrition	Immediate	Other	1
Intermediate	Liver Disease	Immediate	Lower respiratory infections	1
Intermediate	Sepsis	Immediate	Kidney Disease	1
Intermediate	Meningitis/Encephalitis	Immediate	Kidney Disease	1
Intermediate	Meningitis/Encephalitis	Immediate	Diarrheal Diseases	1
Underlying	Other endocrine, metabolic,	Intermediate	Meningitis/Encephalitis	1
Intermediate	Malaria	Immediate	Other	1
Underlying	Congenital infection	Intermediate	Sepsis	1
Underlying	Congenital infection	Intermediate	Meningitis/Encephalitis	1
Intermediate	Lower respiratory infections	Immediate	Other	1
Underlying	Malnutrition	Intermediate	Measles	1
Intermediate	Measles	Immediate	Lower respiratory infections	1
Intermediate	Meningitis/Encephalitis	Immediate	Other	1
Intermediate	Sepsis	Immediate	Other	1
Underlying	Other neurological disorders	Intermediate	Other respiratory disease	1
Underlying	Other neurological disorders	Intermediate	Other	1
Intermediate	Heart Diseases	Immediate	Sepsis	1
Underlying	Congenital infection	Intermediate	Other neonatal disorders	1

© 2023 Bassat Q et al. *JAMA Network Open.*

Underlying	Liver disease	Intermediate	Lower respiratory infections	1
Underlying	Liver disease	Intermediate	Meningitis/Encephalitis	1
Underlying	Liver disease	Intermediate	Liver Disease	1
Intermediate	Liver Disease	Immediate	Sepsis	1
Intermediate	Malnutrition	Immediate	Liver disease	1
Intermediate	Malaria	Immediate	Liver disease	1
Intermediate	Anemias	Immediate	Liver disease	1
Intermediate	Lower respiratory infections	Immediate	Kidney Disease	1
Underlying	Other endocrine, metabolic,	Intermediate	Upper respiratory infections	1
Underlying	HIV	Intermediate	Tuberculosis	1
Underlying	Other neurological disorders	Intermediate	Other infections	1
Intermediate	Anemias	Immediate	Other	1
Intermediate	Other neurological disorders	Immediate	Meningitis/Encephalitis	1
Intermediate	Other	Immediate	Meningitis/Encephalitis	1
Underlying	Neonatal encephalopathy	Intermediate	Other neurological disorders	1
Underlying	Other neurological disorders	Intermediate	Congenital birth defects	1
Intermediate	Other neonatal disorders	Immediate	Lower respiratory infections	1
Underlying	Neonatal encephalopathy	Intermediate	Other	1
Underlying	Neonatal encephalopathy	Intermediate	Other infections	1
Underlying	HIV	Intermediate	Other respiratory disease	1
Underlying	Neonatal preterm birth	Intermediate	Other neurological disorders	1
Underlying	Neonatal preterm birth	Intermediate	Other respiratory disease	1
Underlying	Other neurological disorders	Intermediate	Meningitis/Encephalitis	1
Underlying	Congenital infection	Intermediate	Heart Diseases	1
Underlying	Other neurological disorders	Intermediate	Malnutrition	1
Intermediate	Diarrheal Diseases	Immediate	Other disorders of fluid,	1
Intermediate	Sepsis	Immediate	Other disorders of fluid,	1
Intermediate	Anemias	Immediate	Other disorders of fluid,	1
Underlying	HIV	Intermediate	Liver Disease	1
Intermediate	Other endocrine, metabolic,	Immediate	Sepsis	1

© 2023 Bassat Q et al. *JAMA Network Open.*

Underlying	Malnutrition	Intermediate	Other endocrine, metabolic,	1
Underlying	Diarrheal Diseases	Intermediate	Other neonatal disorders	1
Underlying	Congenital infection	Intermediate	Other respiratory disease	1
Underlying	Congenital infection	Intermediate	Diarrheal Diseases	1
Intermediate	Kidney Disease	Immediate	Meningitis/Encephalitis	1
Intermediate	Other disorders of fluid,	Immediate	Meningitis/Encephalitis	1
Underlying	Sepsis	Intermediate	Neonatal preterm birth	1
Underlying	Sepsis	Intermediate	Other neonatal disorders	1
Intermediate	Neonatal preterm birth	Immediate	Meningitis/Encephalitis	1
Underlying	Congenital infection	Intermediate	Congenital birth defects	1
Underlying	Congenital infection	Intermediate	Other infections	1
Intermediate	Malnutrition	Immediate	Meningitis/Encephalitis	1
Intermediate	Other disorders of fluid,	Immediate	Lower respiratory infections	1
Intermediate	Sepsis	Immediate	Epilepsy	1
Underlying	Other endocrine, metabolic,	Intermediate	Diarrheal Diseases	1
Intermediate	Diarrheal Diseases	Immediate	Epilepsy	1
Intermediate	Other immunodeficiencies	Immediate	Epilepsy	1
Intermediate	Malnutrition	Immediate	Epilepsy	1
Intermediate	Other neurological disorders	Immediate	Epilepsy	1
Underlying	Other endocrine, metabolic,	Intermediate	Other neurological disorders	1
Underlying	Other endocrine, metabolic,	Intermediate	Malnutrition	1
Underlying	Other endocrine, metabolic,	Intermediate	Other immunodeficiencies	1
Underlying	Other endocrine, metabolic,	Intermediate	Sepsis	1

© 2023 Bassat Q et al. *JAMA Network Open.*

eTable 6. Frequencies of causes of death according to place of death (Community vs. facility) and to position in the causal chain (Underlying, Immediate, or all causes pooled together) (sites in Manhiça, Mozambique; Soweto, South Africa; Kisumu and Siaya county, Kenya; Bamako, Mali; Baliakandi, Bangladesh; Makeni, Sierra Leone; Harar and Kersa, Ethiopia), December 2016–December 2020.

	Underlying		Immediate		All[a]		
	Community (N=176) N (%)	Facility (N=456) N (%)	Community (N=176) N (%)	Facility (N=456) N (%)	Community (N=176) N (%)	Facility (N=456) N (%)	p-value[b]
Lower respiratory infections	14 (8.0)	39 (8.6)	29 (16.5)	100 (21.9)	61 (34.7)	219 (48.0)	0.002
Sepsis	9 (5.1)	20 (4.4)	53 (30.1)	138 (30.3)	61 (34.7)	192 (41.5)	0.103
Malnutrition	34 (19.3)	70 (15.4)	2 (1.1)	0 (0)	51 (29.0)	99 (21.7)	0.061
Malaria	21 (11.9)	50 (11.0)	21 (11.9)	23 (5.0)	36 (20.5)	87 (19.1)	0.737
Diarrheal Diseases	9 (5.1)	37 (8.1)	6 (3.4)	20 (4.4)	21 (11.9)	69 (15.1)	0.374
HIV	19 (10.8)	56 (12.3)	0 (0)	1 (0.2)	19 (10.8)	56 (12.3)	0.682
Congenital birth defects	8 (4.5)	56 (12.3)	1 (0.6)	1 (0.2)	8 (4.6)	62 (13.6)	<0.001
Other respiratory disease	8 (4.6)	7 (1.5)	7 (4.0)	29 (6.4)	16 (9.1)	48 (10.5)	0.661
Anemias	1 (0.6)	0 (0)	1 (0.6)	14 (3.1)	3 (1.7)	59 (12.9)	<0.001
Meningitis/Encephalitis	1 (0.6)	5 (1.1)	4 (2.3)	15 (3.3)	9 (5.1)	48 (10.5)	0.043
Other infections	2 (1.1)	9 (2.0)	3 (1.7)	15 (3.3)	8 (4.6)	36 (7.9)	0.164
Neonatal preterm birth complications	7 (4.0)	30 (6.6)	0 (0)	0 (0)	7 (4.0)	35 (7.7)	0.110
Injury	7 (4.0)	24 (5.3)	2 (1.1)	2 (0.4)	9 (5.1)	25 (5.5)	1
Other	3 (1.7)	3 (0.7)	0 (0)	2 (0.4)	10 (5.7)	14 (3.1)	0.161
Other neonatal disorders	1 (0.6)	3 (0.7)	0 (0)	2 (0.4)	1 (0.6)	14 (3.1)	0.080
Other neurological disorders	3 (1.7)	4 (0.9)	0 (0)	0 (0)	4 (2.3)	10 (2.2)	1
Other endocrine, metabolic, blood, and immune disorders	1 (0.6)	4 (0.9)	0 (0)	3 (0.7)	2 (1.1)	9 (2.0)	0.736
Poisoning	2 (1.1)	2 (0.4)	1 (0.6)	3 (0.7)	3 (1.7)	6 (1.3)	0.714
Tuberculosis	0 (0)	5 (1.1)	0 (0)	5 (1.1)	0 (0)	9 (2.0)	0.068
Liver Disease	3 (1.7)	2 (0.4)	1 (0.6)	4 (0.9)	3 (1.7)	9 (2.0)	1
Congenital infection	0 (0)	5 (1.1)	0 (0)	0 (0)	0 (0)	7 (1.5)	0.199
Other skin and subcutaneous diseases	1 (0.6)	2 (0.4)	0 (0)	1 (0.2)	2 (1.1)	5 (1.1)	1
Heart Diseases	0 (0.0)	2 (0.4)	0 (0)	3 (0.7)	0 (0)	6 (1.3)	0.194
Cancer	2 (1.1)	3 (0.7)	0 (0)	1 (0.2)	2 (1.1)	3 (0.7)	0.622
Paralytic ileus and intestinal obstruction	0 (0)	3 (0.7)	0 (0)	0 (0)	0 (0)	5 (1.1)	0.329
Sudden infant death syndrome	5 (2.8)	0 (0)	0 (0)	0 (0)	5 (2.8)	0 (0)	0.002
Epilepsy	0 (0)	2 (0.4)	2 (1.1)	0 (0)	2 (1.1)	2 (0.4)	0.310
Kidney Disease	0 (0)	0 (0)	0 (0)	0 (0)	0 (0)	4 (0.9)	0.580

© 2023 Bassat Q et al. *JAMA Network Open.*

Measles	2 (1.1)	1 (0.2)	0 (0)	0 (0)	3 (1.7)	1 (0.2)	0.068
Neonatal sepsis	0 (0)	1 (0.2)	1 (0.6)	2 (0.4)	1 (0.6)	3 (0.7)	0.356
Other disorders of fluid, electrolyte and acid-base balance	0 (0)	0 (0)	0 (0)	2 (0.4)	0 (0)	4 (0.9)	0.580
Other immunodeficiencies	0 (0)	1 (0.2)	0 (0)	0 (0)	2 (1.1)	2 (0.4)	0.310
Sickle cell disorders	2 (1.1)	0 (0)	1 (0.6)	0 (0)	2 (1.1)	2 (0.4)	0.310
Upper respiratory infections	0 (0)	1 (0.2)	0 (0)	1 (0.2)	0 (0)	3 (0.7)	0.564
Neonatal encephalopathy	0 (0)	1 (0.2)	0 (0)	0 (0)	0 (0)	2 (0.4)	1
Diabetes	0 (0)	1 (0.2)	0 (0)	1 (0.2)	0 (0)	1 (0.2)	1
Motor neuron disease	0 (0)	1 (0.2)	0 (0)	0 (0)	0 (0)	1 (0.2)	1
Other gastrointestinal disease	0 (0)	0 (0)	0 (0)	0 (0)	0 (0)	1 (0.2)	1
Perinatal asphyxia/hypoxia	0 (0)	1 (0.2)	0 (0)	0 (0)	0 (0)	1 (0.2)	1
Rabies	0 (0)	1 (0.2)	0 (0)	0 (0)	0 (0)	1 (0.2)	1

[a] All includes underlying, immediate, and comorbid/intermediate causes of death.

[b] p-values from Fisher's exact test (for the comparison of community vs. facility deaths in all cases).

© 2023 Bassat Q et al. *JAMA Network Open.*

eTable 7. Disease events caused by a given pathogen. Disease-pathogen pairs are sorted by counts (number of occurrences). The first batch of disease-pathogen pairs corresponds to underlying causes. The second batch corresponds to immediate causes.

Stage	Disease	Pathogen	Counts
Underlying	HIV	Human Immunodeficiency Virus	72
Underlying	Malaria	Plasmodium falciparum	70
Underlying	Lower respiratory infections	Streptococcus pneumoniae	20
Underlying	Sepsis	Klebsiella pneumoniae	12
Underlying	Diarrheal Diseases	Escherichia coli	11
Underlying	Lower respiratory infections	Klebsiella pneumoniae	10
Underlying	Sepsis	Other	9
Underlying	Diarrheal Diseases	Other	8
Underlying	Lower respiratory infections	Haemophilus influenzae	7
Underlying	Lower respiratory infections	Other	6
Underlying	Sepsis	Streptococcus pneumoniae	5
Underlying	Other	Mycobacterium tuberculosis	5
Underlying	Lower respiratory infections	Respiratory syncytial virus (RSV)	5
Underlying	HIV	Pneumocystis jirovecii	5
Underlying	Lower respiratory infections	Cytomegalovirus	4
Underlying	Diarrheal Diseases	Rotavirus non-typable	4
Underlying	Other	Cytomegalovirus	4
Underlying	Lower respiratory infections	Adenovirus	4
Underlying	Lower respiratory infections	Staphylococcus aureus	4
Underlying	Lower respiratory infections	Bordetella pertussis	4
Underlying	Diarrheal Diseases	Adenovirus	4
Underlying	Other	Measles morbillivirus	3
Underlying	Sepsis	Cytomegalovirus	3
Underlying	Diarrheal Diseases	Rotavirus A	3
Underlying	Lower respiratory infections	Human metapneumovirus	3
Underlying	Other	Other	3

© 2023 Bassat Q et al. *JAMA Network Open.*

Underlying	Other	Streptococcus pneumoniae	3
Underlying	Lower respiratory infections	Rhinovirus	2
Underlying	Sepsis	Salmonella spp.	2
Underlying	Lower respiratory infections	Pneumocystis jirovecii	2
Underlying	Sepsis	Streptococcus spp.	2
Underlying	Malaria	Other	2
Underlying	Lower respiratory infections	Influenza B Virus	2
Underlying	Diarrheal Diseases	Enterovirus	2
Underlying	Other	Klebsiella pneumoniae	2
Underlying	Sepsis	Escherichia coli/Shigella spp.	1
Underlying	Diarrheal Diseases	Salmonella spp.	1
Underlying	Other	Human Immunodeficiency Virus	1
Underlying	Sepsis	Moraxella catarrhalis	1
Underlying	Sepsis	Adenovirus	1
Underlying	Lower respiratory infections	Influenza A Virus	1
Underlying	Sepsis	Staphylococcus aureus	1
Underlying	Other	Escherichia coli/Shigella spp.	1
Underlying	Lower respiratory infections	Moraxella catarrhalis	1
Underlying	Sepsis	Haemophilus influenzae	1
Immediate	Sepsis	Klebsiella pneumoniae	71
Immediate	Lower respiratory infections	Streptococcus pneumoniae	40
Immediate	Sepsis	Streptococcus pneumoniae	36
Immediate	Lower respiratory infections	Klebsiella pneumoniae	34
Immediate	Sepsis	Escherichia coli	28
Immediate	Lower respiratory infections	Other	27
Immediate	Malaria	Plasmodium falciparum	24
Immediate	Sepsis	Acinetobacter baumannii	18
Immediate	Sepsis	Staphylococcus aureus	18
Immediate	Lower respiratory infections	Cytomegalovirus	18
Immediate	Sepsis	Other	15

© 2023 Bassat Q et al. *JAMA Network Open.*

Immediate	Sepsis	Pseudomonas aeruginosa	15
Immediate	Lower respiratory infections	Staphylococcus aureus	13
Immediate	Sepsis	Haemophilus influenzae	12
Immediate	Lower respiratory infections	Haemophilus influenzae	12
Immediate	Lower respiratory infections	Respiratory syncytial virus (RSV)	11
Immediate	Sepsis	Candida albicans	9
Immediate	Lower respiratory infections	Pneumocystis jirovecii	8
Immediate	Lower respiratory infections	Adenovirus	7
Immediate	Lower respiratory infections	Parainfluenza virus type 3	7
Immediate	Sepsis	Cytomegalovirus	7
Immediate	Diarrheal Diseases	Other	6
Immediate	Lower respiratory infections	Pseudomonas aeruginosa	6
Immediate	Lower respiratory infections	Escherichia coli	6
Immediate	Lower respiratory infections	Influenza A Virus	6
Immediate	Meningitis/Encephalitis	Klebsiella pneumoniae	6
Immediate	Lower respiratory infections	Acinetobacter baumannii	6
Immediate	Sepsis	Candida parapsilosis	5
Immediate	Sepsis	Streptococcus spp.	4
Immediate	Other	Other	4
Immediate	Meningitis/Encephalitis	Streptococcus pneumoniae	4
Immediate	Sepsis	Salmonella spp.	4
Immediate	Sepsis	Escherichia coli/Shigella spp.	4
Immediate	Meningitis/Encephalitis	Other	3
Immediate	Diarrheal Diseases	Escherichia coli	3
Immediate	Sepsis	Enterococcus faecalis	3
Immediate	Sepsis	Streptococcus pyogenes	3
Immediate	Lower respiratory infections	Candida albicans	3
Immediate	Lower respiratory infections	Streptococcus spp.	3
Immediate	Lower respiratory infections	Rhinovirus	3
Immediate	Sepsis	Enterobacter cloacae	3

© 2023 Bassat Q et al. *JAMA Network Open.*

Immediate	Other	Klebsiella pneumoniae	2
Immediate	Sepsis	Adenovirus	2
Immediate	Sepsis	Streptococcus species	2
Immediate	Sepsis	Enterococcus faecium	2
Immediate	Diarrheal Diseases	Enterococcus faecium	1
Immediate	Diarrheal Diseases	Adenovirus	1
Immediate	Lower respiratory infections	Salmonella spp.	1
Immediate	Malaria	Parainfluenza virus type 3	1
Immediate	Diarrheal Diseases	Escherichia coli/Shigella spp.	1
Immediate	Meningitis/Encephalitis	Streptococcus pyogenes	1
Immediate	Diarrheal Diseases	Enterococcus faecalis	1
Immediate	Other	Escherichia coli	1
Immediate	Meningitis/Encephalitis	Cytomegalovirus	1
Immediate	Meningitis/Encephalitis	Streptococcus spp.	1
Immediate	Meningitis/Encephalitis	Haemophilus influenzae	1
Immediate	Lower respiratory infections	Streptococcus species	1
Immediate	Meningitis/Encephalitis	Acinetobacter baumannii	1
Immediate	Meningitis/Encephalitis	Salmonella spp.	1

© 2023 Bassat Q et al. *JAMA Network Open.*

eTable 8. Number of times pathogens were determined to be in the causal chain leading to death. The list includes the 15 most frequently identified pathogens, by condition in causal chain, for deaths among children aged 1 to <59 months, for the CHAMPS Network (sites in Manhiça, Mozambique; Soweto, South Africa; Kisumu and Siaya county, Kenya; Bamako, Mali; Baliakandi, Bangladesh; Makeni, Sierra Leone; Harar and Kersa, Ethiopia), December 2016–December 2020.

**Pathogen**	**HIV (N=75)**	**Malnutrition (N=150)**	**Sepsis (N=253)**	**Diarrhea/ Gastrointestinal syndrome (N=90)**	**Lower respiratory infection (N=280)**	**Meningoencephalitis syndrome or malaria (N=180)**	**Number of deaths in which the pathogen was determined to have been among the causes of death (N=632)**	**Number of deaths in which the pathogen was the only pathogen in the causal chain leading to death**
Klebsiella pneumoniae	17 (22.7)	46 (30.7)	117 (46.2)	23 (25.6)	112 (40.0)	45 (25.0)	155 (24.5)	19 (12.3)
Plasmodium falciparum	12 (16.0)	43 (28.7)	22 (8.7)	5 (5.6)	20 (7.1)	122 (67.8)	122 (19.3)	77 (63.1)
Streptococcus pneumoniae	16 (21.3)	37 (24.7)	62 (24.5)	13 (14.4)	93 (33.2)	23 (12.8)	109 (17.2)	20 (18.3)
HIV	75 (100)	11 (7.3)	31 (12.3)	12 (13.3)	45 (16.1)	17 (9.4)	75 (11.9)	7 (9.3)
Escherichia coli	6 (8.0)	24 (16.0)	42 (16.6)	23 (25.6)	27 (9.6)	15 (8.3)	58 (9.2)	11 (19.0)
Cytomegalovirus (CMV)	20 (26.7)	15 (10.0)	33 (13.0)	3 (3.3)	45 (16.1)	11 (6.1)	57 (9.0)	6 (10.5)
Haemophilus influenzae	7 (9.3)	12 (8.0)	29 (18.3)	6 (6.7)	38 (13.6)	11 (6.1)	46 (7.3)	3 (7.3)
Acinetobacter baumannii	0 (0)	5 (3.3)	35 (13.8)	2 (2.2)	26 (9.3)	11 (6.1)	41 (6.5)	1 (2.6)
Staphylococcus aureus	5 (6.7)	7 (4.7)	31 (12.3)	1 (1.1)	36 (12.9)	5 (2.8)	39 (6.2)	8 (18.6)
Adenovirus	7 (9.3)	4 (2.7)	9 (3.6)	11 (12.2)	16 (5.7)	5 (2.8)	27 (4.3)	7 (25.9)
Pseudomonas aeruginosa	6 (8.0)	6 (4.0)	21 (8.3)	3 (3.3)	19 (6.8)	7 (3.9)	26 (4.1)	1 (3.8)
Pneumocystis jirovecii	12 (16.0)	5 (3.3)	11 (4.3)	3 (3.3)	17 (6.1)	4 (2.2)	22 (3.5)	2 (9.1)
Respiratory syncytial virus (RSV)	3 (4.0)	1 (0.7)	6 (2.4)	1 (1.1)	20 (7.1)	2 (1.1)	21 (3.3)	3 (14.3)
Candida albicans	2 (2.7)	3 (2.0)	11 (4.3)	4 (4.4)	11 (3.9)	3 (1.7)	13 (2.1)	0 (0)
Salmonella spp.	1 (1.3)	6 (4.0)	10 (4.0)	3 (3.3)	5 (1.8)	7 (3.9)	12 (1.9)	5 (38.5)

© 2023 Bassat Q et al. *JAMA Network Open.*

eTable 9: Preventability of studied deaths according to the Decode panel, stratified by age groups and place of death.

	Total (n=632) N (%)	Infant (28 days to <12 months) (N=331) N (%)	Child (12 months to <60 Months) (N=301) N (%)	Community death (N=176) N (%)	In-hospital death (N=456) N (%)
**Preventable**	494 (78.2)	250 (75.5)	244 (81.1)	131 (74.4)	363 (79.6)
**Preventable under certain conditions**	26 (4.1)	18 (5.4)	8 (2.7)	12 (6.8)	14 (3.1)
**Not preventable**	98 (15.5)	53 (16.0)	45 (14.9)	25 (14.2)	73 (16.0)
**No consensus/ Not reported**	14 (2.2)	10 (3.1)	4 (1.3)	8 (4.6)	6 (1.3)

© 2023 Bassat Q et al. *JAMA Network Open.*
