# Supplementary material for: Causes of Death Among Infants and Children in the Child Health and Mortality Prevention Surveillance (CHAMPS) Network
Source: JAMA Netw Open. 2023 Jul 26;6(7):e2322494. doi: 10.1001/jamanetworkopen.2023.22494 (PMC10372710; doi:10.1001/jamanetworkopen.2023.22494)
Supplement: Supplement 2. — Nonauthor Collaborators [file jamanetwopen-e2322494-s002.pdf]

*Group Name(s): Child Health and Mortality Prevention Surveillance (CHAMPS) Network

***First Name and Middle Initial(s)**	***Last Name**	***Suffix (eg, Jr, III)**	Academic Degrees	Institution	Location (city, state/province , country)	Role or Contribution, eg, chair, principal investigator	Group (if more than 1 Group listed in the byline) and/or Subgroup (eg, Steering Committee)
Ferdousi	Begum		MD	Bangabandhu Sheikh Mujib Medical University (BSMMU)	Bangladesh	Data collection	
Mohammad Mosiur	Rahman		MD	Bangabandhu Sheikh Mujib Medical University, Dhaka, Bangladesh	Bangladesh	Data collection	
Mohammed	Kamal		PhD	Bangabandhu Sheikh Mujib Medical University, Dhaka, Bangladesh	Bangladesh	Data collection	
A.S.M. Nawshad Uddin	Ahmed		FCPS	Bangladesh Institute of Child Health at Dhaka University and Dhaka Shishu Children’s Hospital	Bangladesh	Data collection	
Mahbubul	Hoque		FCPS	Bangladesh Institute of Child Health at Dhaka University and Dhaka Shishu Children’s Hospital	Bangladesh	Data collection	
Md. Atique Iqbal	Chowdhury			International Centre for Diarrhoeal Disease Research, Bangladesh (icddr,b)	Bangladesh	Data collection	
Saria	Tasnim		FCPS	Dhaka Community Medical College and Hospital	Bangladesh	Data collection	
Mahbubur	Rahman		MPH	Institute of Epidemiology, Disease Control and Research (IEDCR)	Bangladesh	Data collection	
Tahmina	Shirin		PhD	Institute of Epidemiology, Disease Control and Research (IEDCR)	Bangladesh	Data collection	
Meerjady Sabrina	Flora		PhD	Institute of Epidemiology, Disease Control, and Research (IEDCR), Dhaka, Bangladesh	Bangladesh	Data collection	

*First Name and Middle Initial(s)**	***Last Name**	***Suffix (eg, Jr, III)**	Academic Degrees	Institution	Location (city, state/province, country)	Role or Contribution, eg, chair, principal investigator	Group (if more than 1 Group listed in the byline) and/or Subgroup (eg, Steering Committee)
Dilruba	Ahmed		PhD	International Centre for Diarrhoeal Disease Research, Bangladesh (icddr,b)	Bangladesh	Data collection	
K.	Zaman		PhD	International Centre for Diarrhoeal Disease Research, Bangladesh (icddr,b)	Bangladesh	Data collection	
Mohammed Sabbir	Ahmed		MPH	International Centre for Diarrhoeal Disease Research, Bangladesh (icddr,b)	Bangladesh	Data collection	
Mohammed Zahid	Hossain		PhD	International Centre for Diarrhoeal Disease Research, Bangladesh (icddr,b)	Bangladesh	Data collection	
Mustafizur	Rahman		PhD	International Centre for Diarrhoeal Disease Research, Bangladesh (icddr,b)	Bangladesh	Data collection	
Shahana	Parveen			International Centre for Diarrhoeal Disease Research, Bangladesh (icddr,b)	Bangladesh	Data collection	
Sanwarul	Bari		MD	Maternal and Child Health Division, International Center for Diarrhoeal Diseases Research (icddr,b), Dhaka, Bangladesh	Bangladesh	Data collection	
Qazi Sadek-ur	Rahman		FCPS	International Centre for Diarrhoeal Disease Research, Bangladesh (icddr,b)	Bangladesh	Data collection	
Ferdousi	Islam		FCPS	Popular Medical College and Hospital in Dhaka, Bangladesh	Bangladesh	Data collection	

*First Name and Middle Initial(s)	*Last Name	*Suffix (eg, Jr, III)	Academic Degrees	Institution	Location (city, state/province, country)	Role or Contribution, eg, chair, principal investigator	Group (if more than 1 Group listed in the byline) and/or Subgroup (eg, Steering Committee)
Gutunduru	Revathi		MD	Aga Khan University Hospital, Nairobi, Kenya	Kenya	Data collection	
Aggrey K.	Igunza			Kenya Medical Research Institute, Kisumu	Kenya	Data collection	
Magdalene	Kuria		MMED	Kisumu County Department of Health	Kenya	Data collection	
Paul K.	Mitei		MMED	Kisumu County Department of Health	Kenya	Data collection	
Florence	Murila		MMED	University of Nairobi	Kenya	Data collection	
Jennifer K.	Verani		MD	CDC Atlanta	U.S.A.	Data collection	
Mugah A.	Christopher			Kenya Medical Research Institute, Kisumu	Kenya	Data collection	
Janet	Agaya			Kenya Medical Research Institute, Kisumu	Kenya	Data collection	
Harun O.	Owuor			Kenya Medical Research Institute, Kisumu	Kenya	Data collection	
Gregory O.	Sadat			Kenya Medical Research Institute, Kisumu	Kenya	Data collection	
Joy	Kuboka			US Centers for Disease Control and Prevention, Kisumu	Kenya	Data collection	
Kephas	Otieno			Kenya Medical Research Institute, Kisumu	Kenya	Data collection	
Richard	Oliech			Kenya Medical Research Institute, Kisumu	Kenya	Data collection	

*First Name and Middle Initial(s)	*Last Name	*Suffix (eg, Jr, III)	Academic Degrees	Institution	Location (city, state/province, country)	Role or Contribution, eg, chair, principal investigator	Group (if more than 1 Group listed in the byline) and/or Subgroup (eg, Steering Committee)
Peter O.	Nyamthimba			Kenya Medical Research Institute, Kisumu	Kenya	Data collection	
Aluoch	Anne			Kenya Medical Research Institute, Kisumu	Kenya	Data collection	
Peter O.	Otieno			Kenya Medical Research Institute, Kisumu	Kenya	Data collection	
Dickson	Gethi			Kenya Medical Research Institute, Kisumu	Kenya	Data collection	
Sammy	Khagayi			Kenya Medical Research Institute, Kisumu	Kenya	Data collection	
Joyce Were	Akinyi			Kenya Medical Research Institute, Kisumu	Kenya	Data collection	
Anne	Ogollo			Kenya Medical Research Institute, Kisumu	Kenya	Data collection	
Thomas	Misore			Kenya Medical Research Institute, Kisumu	Kenya	Data collection	
George	Aol			Kenya Medical Research Institute, Kisumu	Kenya	Data collection	
David	Obor			Kenya Medical Research Institute, Kisumu	Kenya	Data collection	
Maryanne	Nyanjom			Kenya Medical Research Institute, Kisumu	Kenya	Data collection	
Mary	Omwalo			Kenya Medical Research Institute, Kisumu	Kenya	Data collection	
Frederick	Omondi			Henry Jackson Foundation Medical Research International (HJFMRI)	Kenya	Data collection	

*First Name and Middle Initial(s)	*Last Name	*Suffix (eg, Jr, III)	Academic Degrees	Institution	Location (city, state/province, country)	Role or Contribution, eg, chair, principal investigator	Group (if more than 1 Group listed in the byline) and/or Subgroup (eg, Steering Committee)
Were Zacchaeus	Abaja			Kenya Medical Research Institute, Kisumu	Kenya	Data collection	
Doh	Sanogo			Centre pour le Développement des Vaccins (CVD-Mali)	Mali	Data collection	
Nana	Kourouma			Centre pour le Développement des Vaccins (CVD-Mali)	Mali	Data collection	
Seydou	Sissoko			Centre pour le Développement des Vaccins (CVD-Mali)	Mali	Data collection	
Uma U.	Onwuchekwu			Centre pour le Développement des Vaccins (CVD-Mali)	Mali	Data collection	
Diakaridia	Sidibe			Centre pour le Développement des Vaccins (CVD-Mali), Ministère de la Santé	Mali	Data collection	
Tatiana	Keita		MD	Clinique Pasteur, Bamako, Mali	Mali	Data collection	
Diakaridia	Kone			CSRef Commune I, Bamako, Mali	Mali	Data collection	
Cheick Bougadari	Traore			Department of pathological Anatlmy and Citology, University Hospital of Point G, Bamako	Mali	Data collection	
Jane	Juma			Centre pour le Développement des Vaccins (CVD-Mali)	Mali	Data collection	
Kounandji	Diarra			Centre pour le Développement des Vaccins (CVD-Mali)	Mali	Data collection	
Awa	Traore			Centre pour le Développement des Vaccins (CVD-Mali)	Mali	Data collection	

***First Name and Middle Initial(s)**	***Last Name**	***Suffix (eg, Jr, III)**	Academic Degrees	Institution	Location (city, state/province, country)	Role or Contribution, eg, chair, principal investigator	Group (if more than 1 Group listed in the byline) and/or Subgroup (eg, Steering Committee)
Tiéman	Diarra			Point Sud, Center for Research on Local Knowledge, Bamako	Mali	Data collection	
Kiranpreet	Chawla			University of Maryland School of Medicine, Baltimore, Maryland, USA	U.S.A.	Data collection	
Sharon M.	Tennant		PhD	University of Maryland School of Medicine, Baltimore, Maryland, USA	U.S.A.	Data collection	
Carol L.	Greene		MD	University of Maryland School of Medicine, Baltimore, Maryland, USA	U.S.A.	Data collection	
J. Kristie	Johnson		PhD	University of Maryland School of Medicine, Baltimore, Maryland, USA	U.S.A.	Data collection	
Rima	Koka		MD	University of Maryland School of Medicine, Baltimore, Maryland, USA	U.S.A.	Data collection	
Karen D.	Fairchild		MD	University of Virginia	U.S.A.	Data collection	
Sandra	Lako		MPH	Aberdeen Women's Centre in Freetown	Sierra Leone	Data collection	
Amara	Jambai			Ministry of Health and Sanitation, Freetown	Sierra Leone	Data collection	
Sartie	Kenneh			Ministry of Health and Sanitation, Freetown	Sierra Leone	Data collection	
Tom	Sesay			Ministry of Health and Sanitation, Freetown	Sierra Leone	Data collection	
Joseph	Bangura			District Health Management Team, Ministry of Health and Sanitation, Bombali District	Sierra Leone	Data collection	
Babatunde	Duduyemi			University of Sierra leone teaching hospital Complex, Freetown	Sierra Leone	Data collection	

*First Name and Middle Initial(s)	*Last Name	*Suffix (eg, Jr, III)	Academic Degrees	Institution	Location (city, state/province, country)	Role or Contribution, eg, chair, principal investigator	Group (if more than 1 Group listed in the byline) and/or Subgroup (eg, Steering Committee)
Princewill Phillip	Nwajiobi			National Hospital, Abuja	Nigeria	Data collection	
Foday	Sessay			District Health Management Team, Ministry of Health and Sanitation, Bombali District	Sierra Leone	Data collection	
Martha	Senessie			Makeni Regional Hospital, Bombali District	Sierra Leone	Data collection	
Chuka	Oham			Makeni Regional Hospital, Bombali District	Sierra Leone	Data collection	
Ibrahim	Bangura			Makeni Regional Hospital, Bombali District	Sierra Leone	Data collection	
Abdulai Othman	Bah			Focus 1000	Sierra Leone	Data collection	
Alim	Swaray-Deen			University of Ghana Medical School	Ghana	Data collection	
Ronita	Luke			Ministry of Health and Sanitation, Freetown	Sierra Leone	Data collection	
Okokon	Ita			University of Calabar teaching Hospital	Nigeria	Data collection	
Cornell	Chukwuegbo			Federal medical Center/PathConsult Diagnostics Ltd., Umuahia	Nigeria	Data collection	
Sulaiman	Sannoh			Division of Neonatology, Department of Pediatrics, St. Luke's University Health Network, Easton PA	U.S.A.	Data collection	

***First Name and Middle Initial(s)**	***Last Name**	***Suffix (eg, Jr, III)**	Academic Degrees	Institution	Location (city, state/province, country)	Role or Contribution, eg, chair, principal investigator	Group (if more than 1 Group listed in the byline) and/or Subgroup (eg, Steering Committee)
Julius	Ojulong			Crown Agents	Sierra Leone	Data collection	
Carrie-Jo	Cain			World Hope International	Sierra Leone	Data collection	
James	Bunn			World Health Organization	Sierra Leone	Data collection	
Hailemariam	Legesse			UNICEF Country Office	Sierra Leone	Data collection	
Francis	Moses			Ministry of Health and Sanitation, Freetown	Sierra Leone	Data collection	
James	Squire			Ministry of Health and Sanitation, Freetown	Sierra Leone	Data collection	
Oluseyi	Balogun			Crown Agents	Sierra Leone	Data collection	
Dickens	Kowuor			Crown Agents	Sierra Leone	Data collection	
Andrew	Moseray			Crown Agents	Sierra Leone	Data collection	
Khadija	Gassama			Crown Agents	Sierra Leone		
Megan	Bias			Infectious Diseases Pathology Branch, Division of High-Consequence Pathogens and Pathology, National Center for emerging and Zoonotic Infectious Diseases, Centers for Disease Control and Prevention, Atlanta, US	U.S.A.	Data collection	

*First Name and Middle Initial(s)	*Last Name	*Suffix (eg, Jr, III)	Academic Degrees	Institution	Location (city, state/province, country)	Role or Contribution, eg, chair, principal investigator	Group (if more than 1 Group listed in the byline) and/or Subgroup (eg, Steering Committee)
Rebecca P.	Philipsborn		MD	Department of Pediatrics, Emory University Hospital, Atlanta, Georgia, USA	U.S.A.	Data collection	
Tais	Wilson			Infectious Diseases Pathology Branch, Division of High-Consequence Pathogens and Pathology, National Center for emerging and Zoonotic Infectious Diseases, Centers for Disease Control and Prevention, Atlanta, US	U.S.A.	Data collection	
Navit T.	Salzberg		MPH	Emory Global Health Institute, Emory University, Atlanta, Georgia	U.S.A.	Data collection	
Jeffrey P.	Koplan		MD	Emory Global Health Institute, Emory University, Atlanta, Georgia	U.S.A.	Data collection	
Josilene Nascimento	Seixas			Infectious Diseases Pathology Branch, Division of High-Consequence Pathogens and Pathology, National Center for emerging and Zoonotic Infectious Diseases, Centers for Disease Control and Prevention, Atlanta, US	U.S.A.	Data collection	

*First Name and Middle Initial(s)	*Last Name	*Suffix (eg, Jr, III)	Academic Degrees	Institution	Location (city, state/province, country)	Role or Contribution, eg, chair, principal investigator	Group (if more than 1 Group listed in the byline) and/or Subgroup (eg, Steering Committee)
Jana M.	Ritter		DVM	Infectious Diseases Pathology Branch, Division of High-Consequence Pathogens and Pathology, National Center for emerging and Zoonotic Infectious Diseases, Centers for Disease Control and Prevention, Atlanta, US	U.S.A.	Data collection	
Margaret	Basket			Emory Global Health Institute, Emory University, Atlanta, Georgia	U.S.A.	Data collection	
Sherif R. (deceased)	Zaki		MD	Infectious Diseases Pathology Branch, Division of High-Consequence Pathogens and Pathology, National Center for emerging and Zoonotic Infectious Diseases, Centers for Disease Control and Prevention, Atlanta, US	U.S.A.	Data collection	
Ashutosh	Wadhwa			Center for Global Health, Centers for Disease Control and prevention, Atlanta	U.S.A.	Data collection	
Jacob	Witherbee			National Center for Immunization and Respiratory Diseases at the Centers for US Disease Control and Prevention	U.S.A.	Data collection	

First Name and Middle Initial(s)	*Last Name	*Suffix (eg, Jr, III)	Academic Degrees	Institution	Location (city, state/province, country)	Role or Contribution, eg, chair, principal investigator	Group (if more than 1 Group listed in the byline) and/or Subgroup (eg, Steering Committee)
Roosecelis	Martines		MD	Infectious Diseases Pathology Branch, Division of High-Consequence Pathogens and Pathology, National Center for emerging and Zoonotic Infectious Diseases, Centers for Disease Control and Prevention, Atlanta, US	U.S.A.	Data collection	
Maureen	Diaz			Respiratory Diseases Branch, Division of Bacterial Diseases, National Center for Immunization and Respiratory Diseases, Centers for Disease Control and Prevention, Atlanta, US	U.S.A.	Data collection	
Jessica	Waller			Respiratory Diseases Branch, Division of Bacterial Diseases, National Center for Immunization and Respiratory Diseases, Centers for Disease Control and Prevention, Atlanta, US			
Jonas M.	Winchell		PhD	Respiratory Diseases Branch, Division of Bacterial Diseases, National Center for Immunization and Respiratory Diseases, Centers for Disease Control and Prevention, Atlanta, US	U.S.A.	Data collection	

*First Name and Middle Initial(s)	*Last Name	*Suffix (eg, Jr, III)	Academic Degrees	Institution	Location (city, state/province, country)	Role or Contribution, eg, chair, principal investigator	Group (if more than 1 Group listed in the byline) and/or Subgroup (eg, Steering Committee)
Joseph O.	Oundo		PhD	1) London School of Hygiene & Tropical Medicine, United Kingdom, 2) College of Health and Medical Sciences, Haramaya University, Harar, Ethiopia	Ethiopia	Decode Panel	
Fikremelekot	Temesgen		MD	Addis Ababa University	Ethiopia	Decode Panel	
Melisachew Mulatu	Yeshi		MD	Ayder Specialized Comprehensive Hospital at Mekelle University	Ethiopia	Decode Panel	
Addisu	Alemu		MD	College of Health and Medical Sciences at Haramaya University	Ethiopia	Decode Panel	
Alexander M.	Ibrahim		MD	College of Health and Medical Sciences at Haramaya University	Ethiopia	Decode Panel	
Tadesse	Gure		MD	College of Health and Medical Sciences at Haramaya University	Ethiopia	Decode Panel	
Stian	Orlien		PhD	University of Hargeisam Somaliland; Vestfold Hospital trust, Tønsberg, Norway	Ethiopia	Decode Panel	
Dadi	Marami			College of Health and Medical Sciences at Haramaya University	Ethiopia	Decode Panel	
Yunus	Edrids		MD	College of Health and Medical Sciences at Haramaya University	Ethiopia	Decode Panel	
Mahlet Abayneh	Gizaw		MD	St. Paul’s Hospital Millennium Medical College in Addis Ababa, Ethiopia	Ethiopia	Decode Panel	
Fentabil	Getnet		PhD	Ethiopian Public Health Institute	Ethiopia	Data collection	

*First Name and Middle Initial(s)	*Last Name	*Suffix (eg, Jr, III)	Academic Degrees	Institution	Location (city, state/province, country)	Role or Contribution, eg, chair, principal investigator	Group (if more than 1 Group listed in the byline) and/or Subgroup (eg, Steering Committee)
Surafel	Fentaw		Mphil	Ethiopian Public Health Institute	Ethiopia	Data collection	
Yasir	Younis			College of Health and Medical Sciences at Haramaya University	Ethiopia	Decode Panel	
Anteneh	Belachew			College of Health and Medical Sciences at Haramaya University	Ethiopia	Decode Panel	
Ayantu	Mekonnen			College of Health and Medical Sciences at Haramaya University	Ethiopia	Decode Panel	
Ephrem	Lemma			College of Health and Medical Sciences at Haramaya University	Ethiopia	Decode Panel	
Henok	Wale			College of Health and Medical Sciences at Haramaya University	Ethiopia	Decode Panel	
Yenework	Acham			Ethiopian Public Health Institute	Ethiopia	Data collection	
Celso	Monjane		MD	Instituto Nacional de Saúde (INS) in Maputo	Mozambique	Data collection	
Sheila	Nhachungue		MD	Instituto Nacional de Saúde (INS) in Maputo	Mozambique	Data collection	
Clara	Menendez		PhD	1) ISGlobal - Hospital Clínic, Universitat de Barcelona; 2) Centro de Investigacao en Saude de Manhica (CISM), Maputo, Mozambique; 3) CIBERESP, Madrid;	Spain	Data collection	
Justina	Bramugy		MD	Centro de Investigacao en Saude de Manhica (CISM), Maputo, Mozambique	Mozambique	Data collection	

*First Name and Middle Initial(s)	*Last Name	*Suffix (eg, Jr, III)	Academic Degrees	Institution	Location (city, state/province, country)	Role or Contribution, eg, chair, principal investigator	Group (if more than 1 Group listed in the byline) and/or Subgroup (eg, Steering Committee)
Milton	Kincardett		MD	Centro de Investigacao en Saude de Manhica (CISM), Maputo, Mozambique	Mozambique	Data collection	
Tacilta	Nhampossa		PhD	Centro de Investigacao en Saude de Manhica (CISM), Maputo, Mozambique; Instituto Nacional Saude, Ministerio Saude, Maputo	Mozambique	Decode Panel	
Ariel	Nhacolo		MSc	Centro de Investigacao en Saude de Manhica (CISM), Maputo, Mozambique	Mozambique	Data collection	
Khátia	Munguambe		PhD	Centro de Investigacao en Saude de Manhica (CISM), Maputo, Mozambique; Eduardo Mondlane University, Faculty of Medicine, Community Health Department, Maputo, Mozambique	Mozambique	Data collection	
Pio	Vitorino		MSc	Centro de Investigacao en Saude de Manhica (CISM), Maputo, Mozambique	Mozambique	Data collection	
Carla	Carrilho		Phd	Department of Pathology, Maputo Central Hospital; Faculty of Medicine, Eduardo Mondlane University, Maputo, Mozambique	Mozambique	Data collection	
Fabiola	Fernandes		PhD	Department of Pathology, Maputo Central Hospital; Faculty of Medicine, Eduardo Mondlane University, Maputo, Mozambique	Mozambique	Data collection	

*First Name and Middle Initial(s)	*Last Name	*Suffix (eg, Jr, III)	Academic Degrees	Institution	Location (city, state/province, country)	Role or Contribution, eg, chair, principal investigator	Group (if more than 1 Group listed in the byline) and/or Subgroup (eg, Steering Committee)
Sozinho	Acácio		PhD	Centro de Investigacao en Saude de Manhica (CISM), Maputo, Mozambique; Instituto Nacional Saude, Ministerio Saude, Maputo	Mozambique	Decode Panel	
Maria	Maixenchs		PhD	ISGlobal Hospital Clinic--Universitat de Barcelona, Spain; Centro de Investigacao en Saude de Manhica (CISM), Manhica, Mozambique	Spain	Data collection	
Juan Carlos	Hurtado		MD	ISGlobal - Hospital Clínic, Universitat de Barcelona	Spain	Decode Panel	
Jaume	Ordi		PhD	ISGlobal - Hospital Clínic, Universitat de Barcelona	Spain	Data collection	
Marta	Valente		MD	ISGlobal - Hospital Clínic, Universitat de Barcelona	Spain	Decode Panel	
Natalia	Rakislova		PhD	ISGlobal - Hospital Clínic, Universitat de Barcelona	Mozambique	Decode Panel	
Dercio	Chitsungo		MD	Quelimane Central Hospital	Mozambique	Data collection	
Zara	Manhique		MD	Quelimane Central Hospital	Mozambique	Data collection	
Elisio	Xerinda		MD	Centro de Investigacao en Saude de Manhica (CISM), Maputo, Mozambique	Mozambique		
Charfudin	Sacoor			Centro de Investigacao en Saude de Manhica (CISM), Maputo, Mozambique	Mozambique		

*First Name and Middle Initial(s)**	***Last Name**	***Suffix (eg, Jr, III)**	Academic Degrees	Institution	Location (city, state/province, country)	Role or Contribution, eg, chair, principal investigator	Group (if more than 1 Group listed in the byline) and/or Subgroup (eg, Steering Committee)
Yasmin	Adam		MD	1) Department of Obstetrics & Gynaecology, Chris Hani Baragwanath Academic Hospital, School of Clinical Medicine, 2) Faculty of Health Sciences, Univeristy of the Witwatersrand, Faculty Health Sciences, Johannesburg, South Africa	South Africa	Data collection	
Sanjay G.	Lala		MD	Department of Paediatrics and Perinatal HIV Research Unit, Chris Hani Baragwanath Academic Hospital, Faculty of Health Sciences, University of the Witwatersrand, Johannesburg, South Africa	South Africa	Data collection	
Karen L.	Petersen		MD	Department of Paediatrics, Chris Hani Baragwanath Academic Hospital, Faculty of Health Sciences, University of the Witwatersrand, Johannesburg, South Africa	South Africa	Data collection	
Peter J.	Swart		MD	National Health for Laboratory Service in South Africa	South Africa	Data collection	
Martin	Hale		MD	National Health Laboratory Service, Department of Anatomical Pathology, School of Pathology, University of the Witwatersrand, Faculty of Health Sciences, Johannesburg, South Africa	South Africa	Data collection	

*First Name and Middle Initial(s)	*Last Name	*Suffix (eg, Jr, III)	Academic Degrees	Institution	Location (city, state/province , country)	Role or Contribution, eg, chair, principal investigator	Group (if more than 1 Group listed in the byline) and/or Subgroup (eg, Steering Committee)
Jeannette	Wadula		MD	National Health Laboratory Service, Department of Microbiology and Infectious Diseases, School of Pathology, University of the Witwatersrand, Faculty of Health Sciences, Johannesburg, South Africa	South Africa	Data collection	
Jeanie	du Toit			South African Council Vaccines and Infectious Diseases Analytics Research Unit, University of Witwatersrand	South Africa	Data collection	
Fatima	Solomon		MD	South African Medical Research Council Vaccines and Infectious Diseases Analytics Research Unit, University of the Witwatersrand, Johannesburg, South Africa	South Africa	Data collection	
Vuyelwa	Baba			University of Witwatersrand, Johannesburg, South Africa	South Africa	Data collection	
Hennie	Lombaard		MD	University of Witwatersrand, Johannesburg, South Africa	South Africa	Data collection	
Nelesh	Govendar			University of Witwatersrand, Johannesburg, South Africa	South Africa	Data collection	
Amy	Wise			University of Witwatersrand, Johannesburg, South Africa	South Africa	Data collection	

***First Name and Middle Initial(s)**	***Last Name**	***Suffix (eg, Jr, III)**	Academic Degrees	Institution	Location (city, state/province, country)	Role or Contribution, eg, chair, principal investigator	Group (if more than 1 Group listed in the byline) and/or Subgroup (eg, Steering Committee)
Constance	Ntuli		Auxillary nurse	South African Council Vaccines and Infectious Diseases Analytics Research Unit, University of Witwatersrand	South Africa	Data collection	
