# Supplementary material for: Causes of Death Among Infants and Children in the Child Health and Mortality Prevention Surveillance (CHAMPS) Network
Source: JAMA Netw Open. 2023 Jul 26;6(7):e2322494. doi: 10.1001/jamanetworkopen.2023.22494 (PMC10372710; doi:10.1001/jamanetworkopen.2023.22494)
Supplement: Supplement 3. — Data Sharing Statement [file jamanetwopen-e2322494-s003.pdf]

Data Sharing Statement

Bassat. Causes of Death Among Infants and Children in the Child Health and Mortality Prevention Surveillance (CHAMPS) Network. *JAMA Netw Open*. Published July 26, 2023. doi:10.1001/jamanetworkopen.2023.22494

Data
Data available: Yes
Data types: Deidentified participant data
How to access data: Data Sharing CHAMPS follows an open approach to data and human sample sharing. Information on CHAMPS data availability can be found at https://champshealth.org. CHAMPS Dataverse: Child Health and Mortality Prevention Surveillance, 2022, "CHAMPS Custom Limited Dataset, v1.0", https://doi.org/10.15139/S3/PBEJDU, UNC Dataverse.
When available: With publication

Supporting Documents
Document types: None

Additional Information
Who can access the data: researchers whose proposed use of the data has been approved
Types of analyses: For any purpose
Mechanisms of data availability: with a signed data access agreement
Any additional restrictions: N/A
